# Centrality of Myeloid-Lineage Phagocytes in Particle-Triggered Inflammation and Autoimmunity

**DOI:** 10.3389/ftox.2021.777768

**Published:** 2021-11-04

**Authors:** Olivia K. Favor, James J. Pestka, Melissa A. Bates, Kin Sing Stephen Lee

**Affiliations:** ^1^ Department of Pharmacology and Toxicology, College of Osteopathic Medicine, Michigan State University, East Lansing, MI, United States; ^2^ Institute for Integrative Toxicology, Michigan State University, East Lansing, MI, United States; ^3^ Department of Food Science and Human Nutrition, Michigan State University, East Lansing, MI, United States; ^4^ Department of Microbiology and Molecular Genetics, Michigan State University, East Lansing, MI, United States; ^5^ Department of Chemistry, Michigan State University, East Lansing, MI, United States

**Keywords:** endogenous and exogenous particles, inflammation, autoimmunity, myeloid-lineage phagocytes, inflammasome activity, immunogenic cell death

## Abstract

Exposure to exogenous particles found as airborne contaminants or endogenous particles that form by crystallization of certain nutrients can activate inflammatory pathways and potentially accelerate autoimmunity onset and progression in genetically predisposed individuals. The first line of innate immunological defense against particles are myeloid-lineage phagocytes, namely macrophages and neutrophils, which recognize/internalize the particles, release inflammatory mediators, undergo programmed/unprogrammed death, and recruit/activate other leukocytes to clear the particles and resolve inflammation. However, immunogenic cell death and release of damage-associated molecules, collectively referred to as “danger signals,” coupled with failure to efficiently clear dead/dying cells, can elicit unresolved inflammation, accumulation of self-antigens, and adaptive leukocyte recruitment/activation. Collectively, these events can promote loss of immunological self-tolerance and onset/progression of autoimmunity. This review discusses critical molecular mechanisms by which exogenous particles (i.e., silica, asbestos, carbon nanotubes, titanium dioxide, aluminum-containing salts) and endogenous particles (i.e., monosodium urate, cholesterol crystals, calcium-containing salts) may promote unresolved inflammation and autoimmunity by inducing toxic responses in myeloid-lineage phagocytes with emphases on inflammasome activation and necrotic and programmed cell death pathways. A prototypical example is occupational exposure to respirable crystalline silica, which is etiologically linked to systemic lupus erythematosus (SLE) and other human autoimmune diseases. Importantly, airway instillation of SLE-prone mice with crystalline silica elicits severe pulmonary pathology involving accumulation of particle-laden alveolar macrophages, dying and dead cells, nuclear and cytoplasmic debris, and neutrophilic inflammation that drive cytokine, chemokine, and interferon-regulated gene expression. Silica-induced immunogenic cell death and danger signal release triggers accumulation of T and B cells, along with IgG-secreting plasma cells, indicative of ectopic lymphoid tissue neogenesis, and broad-spectrum autoantibody production in the lung. These events drive early autoimmunity onset and accelerate end-stage autoimmune glomerulonephritis. Intriguingly, dietary supplementation with ω-3 fatty acids have been demonstrated to be an intervention against silica-triggered murine autoimmunity. Taken together, further insight into how particles drive immunogenic cell death and danger signaling in myeloid-lineage phagocytes and how these responses are influenced by the genome will be essential for identification of novel interventions for preventing and treating inflammatory and autoimmune diseases associated with these agents.

## Introduction

Exogenous and endogenous particles have profound effects on human health. The concept of particle toxicology was first introduced in the 15th century when occupational exposure to dust was etiologically linked to lung disease [reviewed by Donaldson and Seaton (2012)]. Paracelsus, the toxicologist who famously quoted “The dose makes the poison,” documented in a 1567 book his observations of lung disease symptoms in smelters and miners. In 1700, these observations were expanded upon by Bernardino Ramazzini, also known as the father of occupational medicine, who recognized that human disease could be triggered by environmental factors in his work *Diseases of Workers*. Industrialization in the 19th century elicited a rise in occupationally related diseases such as silicosis, asbestosis, lung cancer, and pulmonary fibrosis, leading to a significant increase in both *in vitro* and *in vivo* particle toxicology studies in the 20th century (Perlman and Maier, 2019).

Over the past 50 years, the field of particle toxicology has expanded to encompass not only pathological impacts of environmental particles but also of endogenously formed crystals, hereafter referred to as endogenous particles (Donaldson and Seaton, 2012). Growing interest in endogenous particles is largely attributed to increased worldwide prevalence of genetic hyperuricemia and familial hypercholesterolemia, which are predispositions for crystallization of monosodium urate (MSU) and cholesterol, respectively (Beheshti et al., 2020; Butler et al., 2021). Hyperuricemia is a risk factor for gout, coronary heart disease, and neurodegenerative disorders (Jin et al., 2012; Rock et al., 2013), and hypercholesterolemia is a risk factor for coronary heart disease (Goldstein and Brown, 2015), atherosclerosis (Nidorf et al., 2020), non-alcoholic steatohepatitis (NASH) (Ioannou, 2016), and cholesterol gallstone disease (Di Ciaula et al., 2018). The observed pathological outcomes associated with MSU and cholesterol crystals have spurred ongoing *in vitro* and *in vivo* studies to determine the mechanisms by which these endogenous particles, as well as other types of endogenous particles (e.g., calcium-containing salts) elicit toxicity.

In parallel with the growing interest in particle toxicology, immunologist Polly Matzinger and her colleagues introduced the “danger model” to explain the development of autoimmune disease, which contrasts the classic “self/non-self model” (Matzinger, 1994; Gallucci and Matzinger, 2001; Matzinger, 2002). While the self/non-self model posits that autoreactivity occurs when the adaptive immunity mistakenly recognizes host “self” tissues as foreign “non-self” tissues, the danger model suggests that accumulation of dead cell corpses and released danger signals (e.g., cytokines, chemokines, alarmins, nucleic acids) contribute to heightened proinflammatory responses in innate immune cells, activation of antigen-presenting cells, and differentiation of autoreactive T and B cells, leading to loss of immunological self-tolerance and autoimmunity (Tveita, 2010). In the context of particle toxicology, Matzinger’s danger model provides a useful framework for understanding the mechanisms by which exogenous and endogenous particles induce inflammation and autoimmunity.

The purpose of this review is to provide an overview of critical molecular mechanisms by which exogenous particles (i.e., silica, asbestos, carbon nanotubes, titanium dioxide, aluminum-containing salts) and endogenous particles (i.e., MSU, cholesterol crystals, calcium-containing salts) promote unresolved inflammation and autoimmunity by inducing toxic responses in myeloid-lineage phagocytes with emphases on inflammasome activation and necrotic and programmed cell death pathways. Autoimmune diseases are defined by uncontrolled innate immunity leading to hyperactivation of adaptive immunity, the latter of which drives tissue damage and disease pathogenesis (Doria et al., 2012). The review will focus specifically on myeloid-lineage phagocytes (i.e., macrophages, neutrophils), as these cells comprise the first line of immunological defense against particles (Boraschi et al., 2017).

## Exogenous Particles, Endogenous Particles, and Their Sources

Exogenous particles are defined herein as any particles originating from environmental or synthetic sources. These include silicon dioxide (SiO_2_), asbestos, carbon nanotubes (CNTs), titanium dioxide (TiO_2_), and aluminum-containing salts (alum). SiO_2_ is one of the most abundant compounds in the Earth’s crust (Mossman and Glenn, 2013) and is classified based on its level of crystallinity, with crystalline SiO_2_ (cSiO_2_) demonstrating a periodic order of atoms and amorphous SiO_2_ (aSiO_2_) having either an anarchic order of atoms or crystalline structures (Mulay and Anders, 2016). Asbestos refers to a broad group of fibrous, chain-like silicate minerals that have high tensile strength, large surface area, and resistance to abrasion and chemical corrosion—all characteristics that made it ideal for construction, mining, and other industrial applications such as pipefitting, shipyard work, insulation manufacturing, and textile production in the 20th century (Kusiorowski et al., 2012; Goswami et al., 2013). Like asbestos, CNTs are fibrous, carbon-containing materials that have high tensile strength and large surface area (Iijima 1991), rendering them useful in construction and electronics (Zhang and Lieber, 2016; Venkataraman et al., 2019). TiO_2_ can exist as either nanospheres or nanobelts (Porter et al., 2013), giving them versatile use in construction, agriculture, food additives, cosmetics, and biomedicine (Mohajerani et al., 2019; Baranowska-Wojcik et al., 2020; Musial et al., 2020). Alum was serendipitously discovered as a vaccine adjuvant nearly 100 years ago (Glenny et al., 1926) and is now the most utilized adjuvant in the world (Tomljenovic and Shaw, 2011). Another highly relevant exogenous particle is particulate matter (PM), which may consist of carbon, sulfate, nitrate, silicon, ammonium, and sodium emissions from both manmade and organic sources (Dominici et al., 2015). Due to the complex and heterogenous composition of PM, its toxic mechanisms are much more difficult to characterize than the previously mentioned particles. A detailed discussion of PM toxicity falls outside the scope of this review, but the reader is referred to several excellent reviews on this topic (Adams et al., 2015; Peixoto et al., 2017; Park et al., 2018; Zhao et al., 2019; Kelly and Fussell, 2020; Curtis, 2021).

Exposure to exogenous particles can occur by inhalation, ingestion, or injection. SiO_2_ was first identified as an inhalation hazard in the 1920s when it was etiologically linked to silicosis in miners (Elliott, 1923; Heffernan, 1929). Today, SiO_2_ remains an occupational inhalation hazard in construction, mining, ceramic manufacturing, dental mold production, and jewelry production (Fazen et al., 2020; Hall et al., 2020; Russ et al., 2020). Asbestos exposure primarily occurs by inhalation (Mossman et al., 2011), and despite decreased industrial use in the United States and Europe, industrial asbestos use is being deferred to Asian and Latin-American countries (Pira et al., 2018). CNTs can either pose as respirable toxicants similar to asbestos fibers in industrial settings (Donaldson et al., 2010) or function as carrier systems in targeted drug, vaccine, cancer, and gene therapies (Beg et al., 2011; Negri et al., 2020). TiO_2_ exposure can occur by inhalation in industrial environments or ingestion of commercial products, and it exhibits toxicity in the lungs, digestive tract, brain, and cardiovascular system (Baranowska-Wojcik et al., 2020). Exposure to alum occurs primarily by injection as a vaccine adjuvant (Gherardi and Authier, 2012) but can also occur by inhalation in foundry work and related occupations (Rifat et al., 1990; Polizzi et al., 2002). While the National Institute for Occupational Safety and Health (NIOSH) recommends using respirators in occupations with high, prolonged particle exposure (NIOSH, 2019), low compliance with such guidelines is associated with respirator discomfort, lack of training on health hazards, self-employment, and breathing problems that would be aggravated by respirator use (Fukakusa et al., 2011).

Endogenous particles are defined as any particles that form within biological systems. From an environmental perspective, many of these are formed by crystallization of nutrients, typically in individuals with corresponding genetic predispositions. Endogenous particles include MSU, cholesterol crystals (CCs), and calcium salts such as calcium phosphate (CaP) and calcium oxalate (CaOx). MSU originates from crystallized uric acid, a byproduct of purine nucleic acid catabolism released by dying cells (Gallo and Gallucci, 2013). Cholesterol is derived from dietary sources and biosynthesis in the liver (Pownall and Gotto, 2019). Dysregulated cholesterol metabolism can contribute to deposition of low-density lipoproteins (LDLs) and high-density lipoproteins (HDLs) in tissues, engulfment of LDLs and HDLs by recruited macrophages and dendritic cells (DCs), and intracellular CC formation (Schaftenaar et al., 2016; Defesche et al., 2017; Varsano et al., 2018). Like cholesterol, calcium occurs both in dietary and body sources, and it can crystallize as CaP and CaOx salts within renal tubules and blood vessels (Khan et al., 2016; Karasawa and Takahashi, 2017). While biomolecules and minerals found in endogenous particles can originate from diet and/or metabolism, crystal formation itself occurs in myeloid phagocytes and along tubular structures within the body.

Endogenous particles are thought to form by crystallization resulting from supersaturation of biological molecules (e.g., cholesterol, uric acid) and minerals (e.g., calcium) in the joints, arteries, and urinary tract (Mulay and Anders, 2016). Although the precise mechanisms for crystal formation have yet to be elucidated, genome-wide associated studies have identified loci that contribute to overproduction and insufficient metabolism of uric acid, LDL, HDL, and calcium-containing salts (Holdt and Teupser, 2013; Dron and Hegele, 2017; Sayer, 2017; Kawamura et al., 2019; Tai et al., 2019). Overabundance of these biomolecules in synovial fluid, serum, or urine creates conditions for supersaturation, increasing the likelihood of crystallization and disease development (Table 1).

**TABLE 1:** Sources and common exposure routes of exogenous and endogenous particles.

Name	Sources	Route	References
SiO_2_	Construction, mining, ceramic manufacturing, dental mold production, jewelry production	Inhalation	Fazen et al. (2020); Russ et al. (2020); Hall et al. (2020)
Asbestos	Construction, mining, pipefitting, shipyard work, insulation manufacturing, textile production	Inhalation	Mossman et al. (2011); Kusiorowski et al. (2012); Goswami et al. (2013)
CNTs	Construction, electronics, biomedicine	Inhalation, Injection	Donaldson et al. (2010); Beg et al. (2011); Zhang and Lieber (2016); Negri et al. (2020)
TiO_2_	Manufacturing, agriculture, food additives, cosmetics, biomedicine	Inhalation, Ingestion	Mohajerani et al. (2019); Baranowska-Wojcik et al. (2020); Musial et al. (2020)
Alum	Foundry work, vaccine adjuvants	Inhalation, Injection	Polizzi et al. (2002); Tomljenovic and Shaw (2011); Rifat et al. (1990); Gherardi and Authier (2012)
MSU	Dietary uric acid, dysregulated purine metabolism, hyperuricemia	N/A	Holdt and Teupser (2013); Dron and Hegele (2017); Sayer (2017); Tai et al. (2019); Kawamura et al. (2019);
CCs	Dietary cholesterol, dysregulated cholesterol metabolism, hypercholesterolemia	N/A	Schaftenaar et al. (2016); Defesche et al. (2017); Pownall and Gotto (2019); Dron and Hegele (2017)
CaP	Dietary calcium and phosphate, hypercalcituria, hyperphosphatemia	N/A	Khan et al. (2016); Karasawa and Takahashi (2017); Sayer (2017)
CaOx	Dietary calcium and oxalate, hypercalcituria, hyperoxaluria	N/A	Khan et al. (2016); Karasawa and Takahashi (2017); Sayer (2017);

Alum, aluminum-containing salts; CaOx, calcium oxalate; CaP, calcium phosphate; CCs, cholesterol crystals; CNTs, carbon nanotubes; MSU, monosodium urate; SiO_2_, silicon dioxide; TiO_2_, titanium dioxide.

## Recognition of Exogenous and Endogenous Particles by Myeloid-Lineage Phagocytes

**FIGURE 1:**
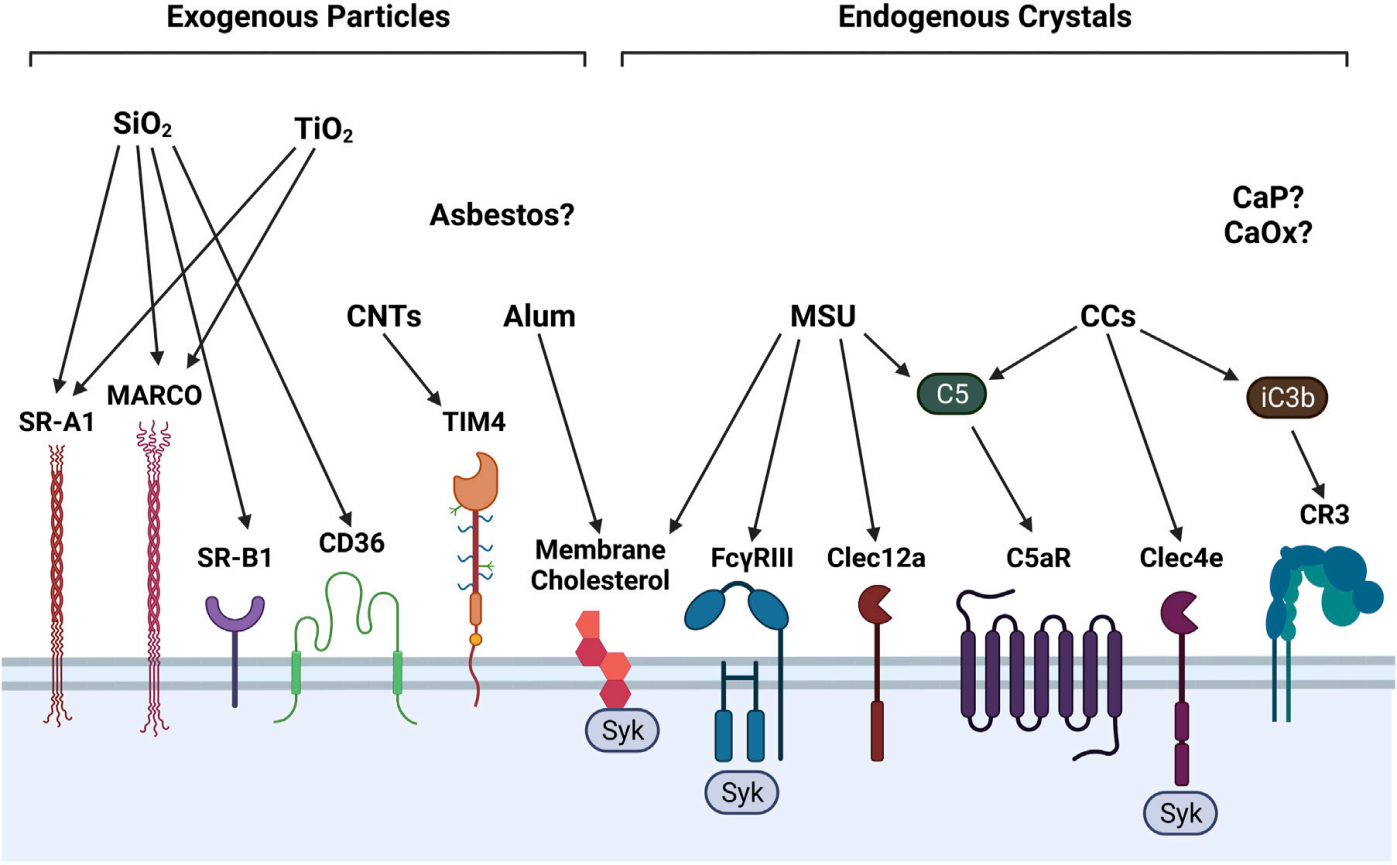
Surface receptors involved in detecting exogenous and endogenous particles. Phagocytes employ a diverse assortment of membrane receptors to recognize and ultimately phagocytose particles, some of which are depicted in this illustration. SiO_2_ is recognized by scavenger receptors SR-A1, MARCO, SR-B1, CD36. TiO_2_ is recognized only by SR-A1 and MARCO. CNTs are recognized by phosphatidylserine receptor Tim4. Alum and MSU interact directly with membrane cholesterol moieties to stimulate Syk signaling. MSU and CCs activate complement components C5 and iC3b, which stimulate C5aR and CR3, respectively. MSU also binds to FcγRIII/CD16 and C-type lectin (Clec)-12a. On human phagocytes only, CCs are recognized by Clec4e. Surface receptors for asbestos fibers and calcium-containing salts (e.g., CaP, CaOx) have not yet been identified.

Particles can stimulate multiple types of surface receptors to promote incorporation into phagosomes, or intracellular vesicles that transport phagocytosed particles. Macrophages, neutrophils, and DCs can recognize particles through a diverse repertoire of surface receptors (Figure 1). For instance, SiO_2_ and TiO_2_ both bind to members of the class A scavenger receptor family including SR-A1 and macrophage receptor with collagenous structure (MARCO). However, SiO_2_ also binds the class B scavenger receptors SR-B1 and CD36/SR-B2, whereas TiO_2_ does not (Thakur et al., 2009; Tsugita et al., 2017b; Nishijima et al., 2017). In macrophages, stimulation of class A and class B scavenger receptors by their respective ligands has been associated with p38 MAPK and JNK activation and enhanced particle endocytosis (Zani et al., 2015). Alternatively, CNTs, which are more fibrous than SiO_2_ and TiO_2_ particles, are recognized by the phosphatidylserine receptor T cell immunoglobulin mucin 4 (Tim4) (Omori et al., 2021).

Contrary to exogenous particles, endogenous particles are recognized by a more diverse set of surface receptors and elicit different intracellular signaling pathways. For example, MSU crystals interact with C-type lectin (Clec)-12a on macrophages and DCs (Neumann et al., 2014; Li et al., 2019a) and FcγRIII/CD16 on neutrophils (Barabe et al., 1998). FcγRIII is also expressed in murine macrophages and DCs (Nimmerjahn and Ravetch, 2008). On human macrophages, neutrophils, and DCs, CCs can bind to Clec4e to potentiate proinflammatory immune responses (Kiyotake et al., 2015). FcγRIII stimulation by MSU and Clec4e stimulation by CCs trigger downstream spleen tyrosine kinase (Syk) signaling (Desaulniers et al., 2001; Clement et al., 2016). Alternatively, both MSU and alum can directly interact with membrane cholesterol moieties and induce Syk signaling in DCs, potentially by lipid membrane sorting (Ng et al., 2008; Flach et al., 2011).

Surface receptors for asbestos, CaP, and CaOx have not yet been identified, but it is possible that phagocytes recognize these particles directly by membrane lipid binding or indirectly through complement receptor signaling. Accordingly, complement C5 binding to the C5a receptor (C5aR) can amplify MSU-driven toxicity (An et al., 2014). In addition, activation of C5aR by C5 and complement receptor 3 (CR3) by complement factor iC3b can augment CC-induced toxic responses (Niyonzima et al., 2017).

### Differential Expression of Particle-Sensing Receptors in Myeloid-Lineage Phagocytes

Not only is it important to consider the types of surface receptors that can be stimulated by particles, but it is also crucial to further emphasize which myeloid-lineage phagocytes express which receptors, because different particles might activate different subsets of myeloid cells. For instance, SR-A1 is expressed by macrophages, monocytes, and DCs, while MARCO is primarily expressed by macrophages and DCs (Ingersoll et al., 2010; Stephen et al., 2010). CD36 is expressed by many cell types including macrophages, monocytes, DCs, and non-hematopoietic cells, whereas SR-B1 is predominantly expressed by macrophages and hepatocytes (Prabhudas et al., 2014; Li et al., 2020). Macrophages and DCs have been shown to express Tim4, but data pertaining to Tim4 expression in neutrophils is currently lacking (Wong et al., 2010; Caronni et al., 2021). On the other hand, macrophages, neutrophils, and DCs express Clec12a (Neumann et al., 2014; Li et al., 2019a; Vaillancourt et al., 2021), FcγRIII (Barabe et al., 1998; Nimmerjahn and Ravetch, 2008), and Clec4e (in humans only) (Kiyotake et al., 2015). Collectively, these observations suggest that myeloid-lineage phagocytes might be better prepared to respond to endogenous particles compared to exogenous particles. Nevertheless, additional research is required to confirm or reject such a hypothesis.

Several studies published over the past decade have shed additional light on differential expression patterns of particle-sensing receptors in tissue-resident macrophages that commonly interact with particles, including bone marrow-derived macrophages (BMDMΦs), alveolar macrophages (AMΦs), and hepatic Kupffer cells (KCs). A comprehensive gene expression review across different tissue-resident macrophage types found that SR-A1 expression is high in BMDMΦs and low in both AMΦs and KCs, whereas MARCO expression is low in BMDMΦs and high in both AMΦs and KCs (Ley et al., 2016). In the same analysis, notable observations were made in relation to the other receptors mentioned in the present review: 1) SR-B1 expression is higher in AMΦs and KCs compared to BMDMΦs; 2) CD36 expression is high in BMDMΦs but lower in AMΦs and KCs; 3) Tim4 expression is low in BMDMΦs and AMΦs but high in KCs; 4) Clec12a is highly expressed in BMDMΦs but not in AMΦs or KCs; 5) Clec4e expression is high in BMDMΦs and AMΦs but low in KCs; 6) FcγRIII is highly expressed in BMDMΦs, AMΦs, and KCs; and 7) C5aR expression is high only in BMDMΦs (Ley et al., 2016). In two different studies, MARCO and Tim4 expression were found to be lower in BMDMΦs compared to KCs (Beattie et al., 2016; A-Gonzalez et al., 2017). Two other studies also showed that Clec4e expression increases in macrophages localized to the kidneys during acute renal inflammation, suggesting Clec4e perpetuates proinflammatory cytokine signaling and cell death in the kidney (Lv et al., 2017; Tanaka et al., 2020).

Not only do tissue-resident macrophages demonstrate differential expression patterns for many particle-sensing surface receptors, but similar patterns can be detected in blood-derived monocytes. A single-cell gene expression analysis with human monocytes found that expression levels for SR-A1, MARCO, CD36, and Clec4e significantly differed between classical monocytes (CD14^++^CD16^–^), intermediate monocytes (CD14^++^CD16^+^), and non-classical monocytes (CD14^+^CD16^++^) (Gren et al., 2015). A different study comparing FcγRIII expression in classical and non-classical monocytes found that expression was higher in classical monocytes than non-classical monocytes in mice, but expression was lower in classical monocytes than non-classical monocytes in humans (Kerntke et al., 2020). Furthermore, FcγRIII expression in murine classical monocytes was similar to that in neutrophils, while expression in human neutrophils was remarkably higher than both classical and non-classical monocytes (Kerntke et al., 2020). Although surface receptor expression patterns were not compared between monocytes and macrophages in either of these studies, such distinctions might require a case-by-case basis approach. For instance, monocytes and BMDMΦs express similar levels of Clec12a (Lobato-Pascual et al., 2013), but CD36 expression increases in monocytes differentiating into BMDMΦs (Huh et al., 1996). Accordingly, future research in this area would provide valuable insight into specific myeloid-lineage phagocyte subsets that respond to different types of exogenous and endogenous particles. Future therapies for particle-induced inflammatory and autoimmune diseases may potentially include antagonists that prevent particle-receptor interactions and downstream toxicity.

## Inflammasome Activation: A Central Mechanism of Particle-Induced Toxicity and Proinflammatory Immune Responses

Following phagocytosis, one central mechanism of toxicity initiated by exogenous and endogenous particles alike is inflammasome activation (Martinon et al., 2006; Dostert et al., 2008; Hornung et al., 2008; Tsugita et al., 2017a; Dominguez-Gutierrez et al., 2018; Karasawa and Takahashi, 2019; Luz et al., 2019). Inflammasomes are cytosolic multiprotein complexes that assemble upon sensing diverse stimuli—including microbial moieties, endogenous danger signals, and particles—to promote proinflammatory signaling (Strowig et al., 2012; Sayan and Mossman, 2016). Because of their importance in orchestrating innate immune responses, inflammasomes are primarily studied in innate immune cells, most notably macrophages; however, other investigators are beginning to investigate their roles in adaptive immune cells and nonhematopoietic cells (Gasteiger et al., 2017). Pattern recognition receptors (PRRs) from the nucleotide-binding oligomerization domain (NOD) leucine-rich region-containing receptor (NLR) family, including NLRP1, NLRP3, and NLRC4, as well as absent-in-melanoma 2 (AIM2) and pyrin, form well-defined inflammasome complexes (Haneklaus and O’Neill, 2015; Duncan and Canna, 2018; Heilig and Broz, 2018; Lugrin and Martinon, 2018; Mitchell et al., 2019). In addition, the NLRs NLRP2, NLRP6, NLRP7, NLRP12, and NLRC5, as well as interferon-inducible protein 16 (IFI16), form inflammasome complexes, albeit less well-characterized or atypical complexes (Elinav et al., 2011; Khare et al., 2012; Vladimer et al., 2012; Janowski and Sutterwala, 2016; Matsuoka et al., 2019).

The NLRP3 inflammasome is the most studied inflammasome due to its putative roles in various pathologies including rheumatic disease (So et al., 2013), Alzheimer’s disease (Heneka et al., 2018), acute myocardial infarction (Toldo and Abbate, 2018), kidney disease (Komada and Muruve, 2019), type 2 diabetes (Wada and Makino, 2016), obesity (Rheinheimer et al., 2017), cancer (Moossavi et al., 2018), and COVID-19, which is caused by severe acute respiratory syndrome coronavirus 2 (SARS-CoV-2) infection (Freeman and Swartz, 2020). This inflammasome also plays a pertinent role in particle-driven diseases such as pulmonary fibrosis, asthma, chronic obstructive pulmonary disease (COPD), malignant mesothelioma, and other lung cancers (Sayan and Mossman, 2016). NLRP3 inflammasome oligomers consist of the NOD-like receptor NLRP3, the adapter protein apoptosis-associated speck-like protein containing a caspase recruitment domain (ASC), and pro-caspase-1 as an effector (Kelley et al., 2019). Three distinct pathways are implicated for NLRP3 inflammasome activation: 1) the canonical pathway, 2) the alternative pathway, and 3) the noncanonical pathway (He et al., 2016). The alternative and noncanonical pathways fall beyond the scope of this review, though readers are directed to other excellent discussions of these topics for further information (Gaidt and Hornung, 2017; Yi, 2017).

### Step 1: Priming

**FIGURE 2:**
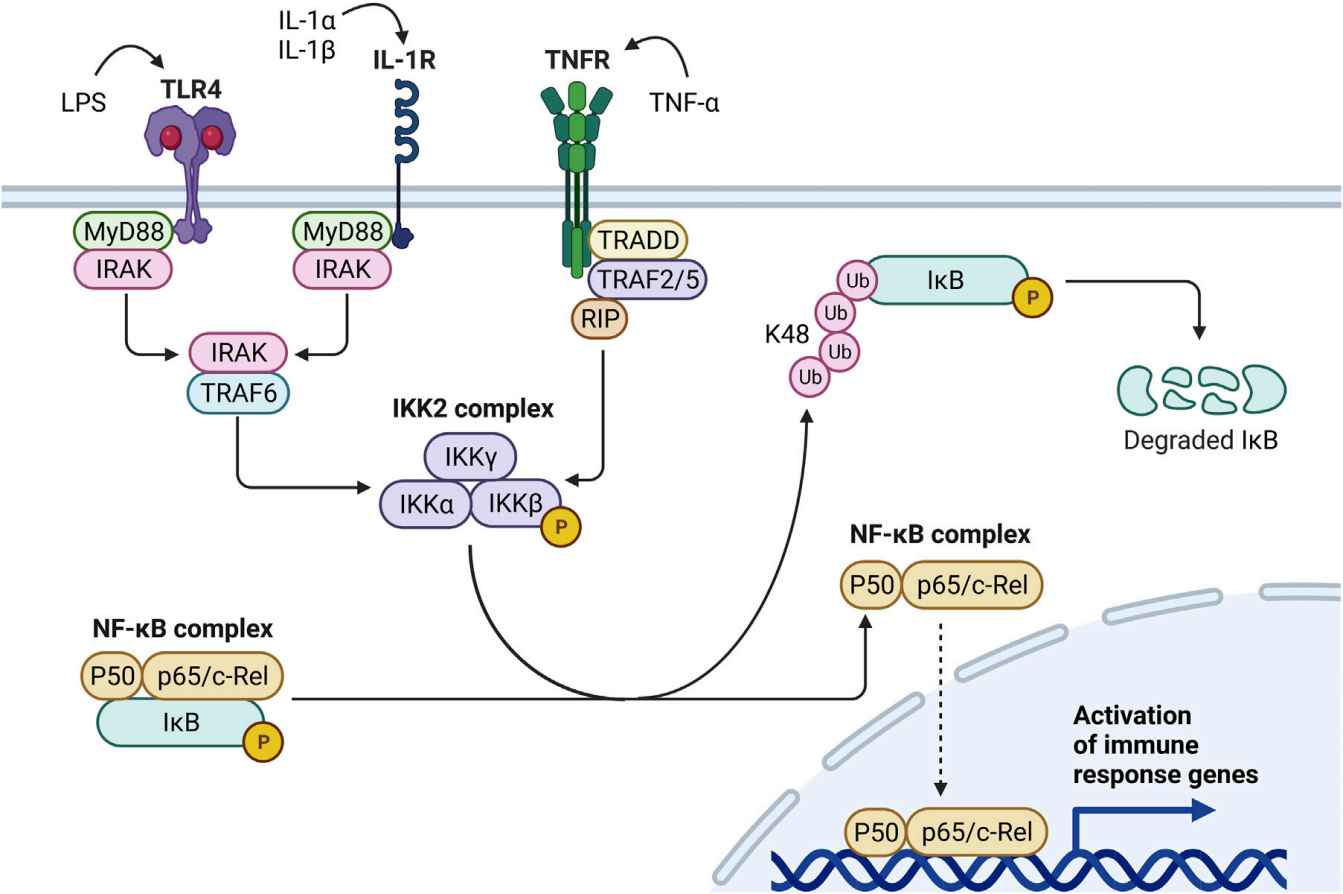
Mechanisms of Signal 1 inflammasome priming. Inflammasome priming can be triggered by diverse stimuli including bacterial molecules (e.g., LPS), alarmins (e.g., IL-1α), or proinflammatory cytokines (e.g., IL-1β, TNF-α). LPS binds to TLR4, activates the MyD88-IRAK-TRAF6 pathway, and induces IKKβ activity within the IKK2 complex. Likewise, by binding IL-1R, IL-1α and IL-1β promote IKKβ activity through the MyD88-IRAK-TRAF6 pathway. Conversely, when TNF-α binds TNFR, the TRADD-TRAF2/5-RIP pathway induces IKKβ activity. Once activated, IKKβ phosphorylates IκB within the NF-κB complex, targeting IκB for K48 polyubiquitination and proteasomal degradation. IκB degradation liberates the NF-κB complex (i.e., P50 and p65/c-Rel) and enables its translocation to the nucleus, where it upregulates proinflammatory cytokines, chemokines, and other immune response genes.

Canonical inflammasome activation occurs in a two-step process that first requires a priming signal to promote transcriptional upregulation of inflammasome-related proteins and a subsequent activating signal to trigger inflammasome oligomerization and caspase-1 activation (Zheng et al., 2020). Priming can be accomplished upon recognition of damage-associated molecular patterns (DAMPs), pathogen-associated molecular patterns (PAMPs), or cytokines by specific surface receptors. For example, the bacterial PAMP lipopolysaccharide (LPS) activates toll-like receptor (TLR)-4, the endogenous DAMP high group mobility group box 1 (HMGB1) activates TLR2/4/9, and tumor necrosis factor (TNF)-α and interleukin (IL)-1α activate the TNF and IL-1 receptors, respectively (Rai and Agrawal, 2017; McKee and Coll, 2020; Wang and Zhang, 2020). These binding events contribute to phosphorylation of the inhibitor of nuclear factor kappa-B kinase (IKK)-β subunit within the cytosolic IKK2 complex. IKKβ then phosphorylates IκBα and targets it for K48-ubiquitination and proteasomal degradation. Degradation of IκBα liberates the dimeric transcription factor nuclear factor-kappa B (NF-κB), allowing its translocation into the nucleus where it upregulates the inflammasome subunits NLRP3, ASC, and pro-caspase-1 as well as pro-IL-1β and pro-IL-18 (Dorrington and Fraser, 2019) (Figure 2). Under homeostatic conditions, DAMPs and proinflammatory cytokines are typically contained inside phagocytes; however, these danger signals can be released into the extracellular environment following particle-induced cell death (Zitvogel et al., 2010; Rabolli et al., 2016). If clearance of extracellular particles, DAMPs, and cytokines is hindered, perpetual stimulation of DAMP/cytokine receptors and particle-sensing receptors may ensue, leading to aberrant inflammasome priming and activation.

### Step 2: Activation

**FIGURE 3:**
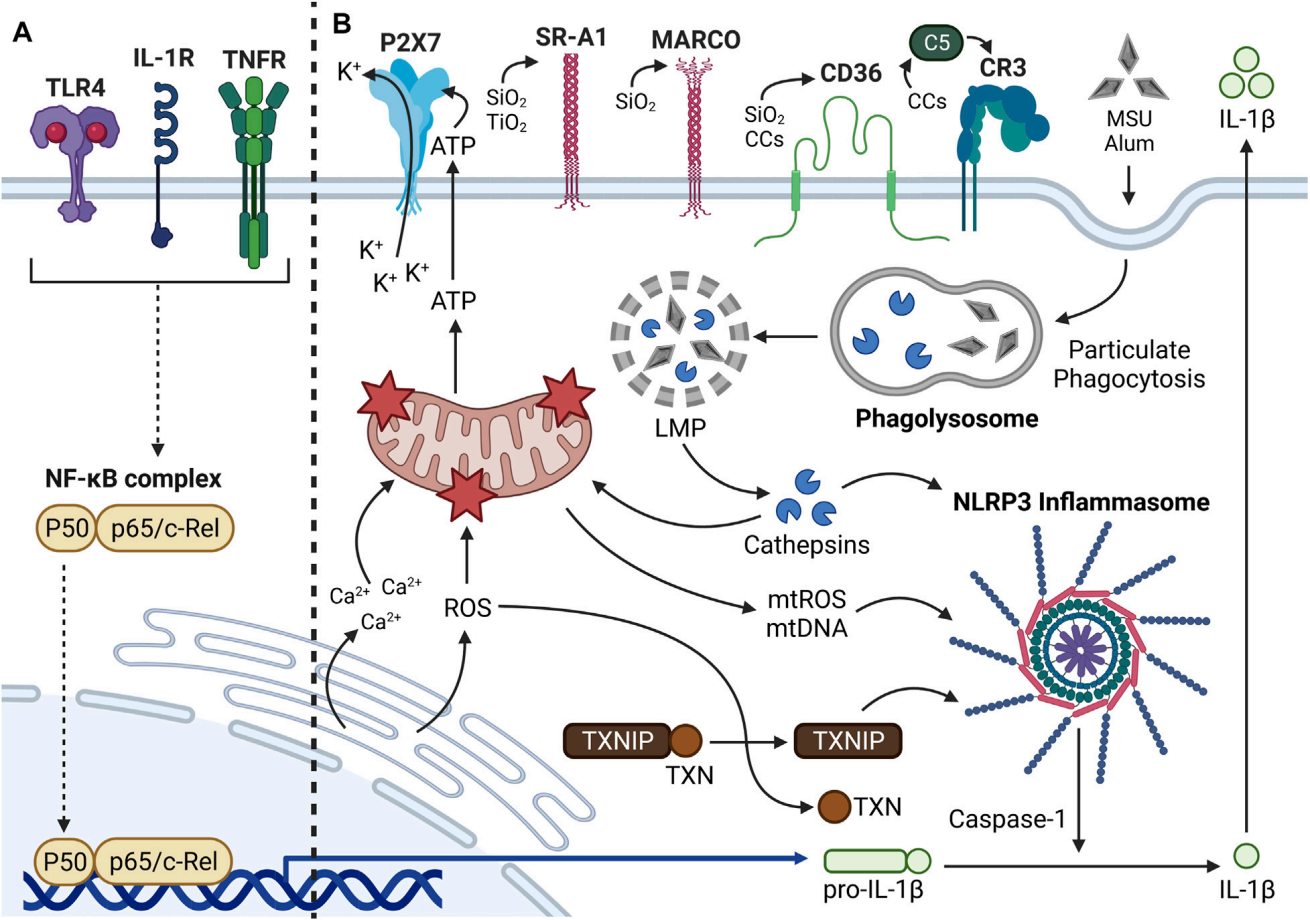
Mechanisms of Signal 2 inflammasome activation. **(A)** Summary of Signal 1 inflammasome priming. Translocation of NF-κB into the nucleus leads to upregulation of proinflammatory cytokines such as pro-IL-1β and inflammasome subunits (i.e., NLRP3, ASC, pro-caspase-1; not shown). **(B)** The NLRP3 inflammasome is a cytosolic multiprotein complex that promotes proinflammatory cytokine production in response to extracellular stimuli and intracellular stress. Many extracellular and intracellular components can be involved in particle-driven inflammasome oligomerization and activity. Some particles (e.g., SiO_2_, TiO_2_, CCs) bind transmembrane receptors prior to phagocytosis, whereas other particles (e.g., MSU, alum) interact directly with the plasma membrane. Following phagocytosis, the particle-containing phagosomes fuse with a lysosome to form a phagolysosome. Through undefined mechanisms, the particles aggravate the phagolysosomal membrane and induce lysosomal membrane permeabilization (LMP), which causes release of lysosomal proteases called cathepsins into the cytosol. Some cathepsins such as cathepsin B can directly trigger inflammasome oligomerization. Cathepsins can cause mitochondrial dysfunction and release of mtDAMPs (e.g., ATP, mtROS, mtDNA) into the cytosol. ATP released from dying phagocytes can interact with P2X7 receptors and trigger K^+^ efflux, which can contribute to inflammasome activation. mtROS and mtDNA can also contribute significantly to inflammasome oligomerization. Mitochondrial dysfunction can alternatively be elicited by CHOP-mediated Ca^2+^ release and ROS production from the ER. Cytosolic ROS contributes to dissociation of TXN from TXNIP, the latter of which can promote inflammasome activation. Once the inflammasome is assembled, pro-caspase-1 proteolytically activates adjacent pro-caspase-1 moieties. Activated caspase-1 then proteolytically processes pro-IL-1β to IL-1β, which is ultimately released from the cell to interact with IL-1R on neighboring phagocytes.

Following the priming signal, a separate activating signal triggers inflammasome assembly and caspase-1 maturation. Contrary to the priming step, which is initiated by a select set of ligands, the activating step can be triggered by many different stimuli including ATP (Di Virgilio et al., 2017), mitochondrial reactive oxygen species (mtROS) (Zhou et al., 2011), mitochondrial DNA (mtDNA) (Shimada et al., 2012), ceramide (Scheiblich et al., 2017), bacterial toxins (Munoz-Planillo et al., 2013), and particles (Sayan and Mossman, 2016). The diverse nature of these stimuli suggests they do not directly act upon inflammasome subunits but rather induce a few common intracellular events that lead to inflammasome oligomerization. Lawlor and Vince propose that these signals may converge on lysosomal rupture, mitochondrial dysfunction, and endoplasmic reticulum (ER) stress (Lawlor and Vince, 2014) (Figure 3).

#### Lysosomal Membrane Permeabilization

Once particles or other danger signals are incorporated into a phagosome, the phagosome fuses with a lysosome to form an intracellular phagolysosome (Rosales and Uribe-Querol, 2017). The role of the phagolysosome is to digest internalized materials; however, many crystalline particles such as cSiO_2_, CCs, alum, and MSU disrupt the phagolysosomal membrane in a process called lysosomal membrane permeabilization (LMP) (Hornung et al., 2008; Campden and Zhang, 2019). LMP describes any process by which the lysosomal membrane is disrupted and lysosomal enzymes including cathepsins are released into the cytosol (Boya and Kroemer, 2008). Although the precise mechanisms by which particles induce LMP remain unknown, one critical study recently found that a subfamily of silanols, termed “nearly free silanols,” on the surface of cSiO_2_ and aSiO_2_ particles promote membranolysis by direct membrane interaction (Pavan et al., 2020). Once cathepsins are released from ruptured phagolysosomes, some cathepsins may directly activate the inflammasome (Niemi et al., 2011; Orlowski et al., 2015; Chevriaux et al., 2020) or elicit dysfunction of other intracellular organelles, including the mitochondria and ER, that can indirectly activate the inflammasome. Accordingly, exogenous and endogenous particles that are engulfed by phagocytes can directly elicit LMP and indirectly promote mitochondrial and ER stress.

#### Mitochondrial Dysfunction

As mentioned in the previous section, cathepsins released by particle-triggered LMP may promote downstream mitochondrial dysfunction (Thibodeau et al., 2004; Joshi and Knecht, 2013). Mitochondrial dysfunction has been linked to inflammasome activation specifically by the release of mitochondrial DAMPs (mtDAMPs) such as ATP, oxidized mtDNA, and mtROS (Zhou et al., 2011; Mills et al., 2017). A large body of evidence suggests ATP can trigger inflammasome assembly and caspase-1 activation in macrophages, specifically by promoting K^+^ ion efflux through either the P2X7 surface receptor or the TWIK2 K^+^ channel (Luna-Gomes et al., 2015; Di Virgilio et al., 2017; Martinez-Garcia et al., 2019). In phagocytes, particle exposure also can trigger apoptosis, a process that can begin in the mitochondria (Hamilton et al., 1996; Iyer et al., 1996; Lim et al., 1999; Thibodeau et al., 2003; Hu et al., 2006; Hirano et al., 2017). It is possible that opening of the mitochondrial permeability transition pore (MPTP) during apoptosis allows oxidized mtDNA and mtROS to exit depolarized mitochondria and activate the inflammasome, but this requires additional investigation. Once in the cytosol, oxidized mtDNA can directly bind NLRP3 to promote caspase-1 activation and resultant IL-1β maturation (Shimada et al., 2012; Zhong et al., 2018).

Conversely, the requirement of mtROS in inflammasome activation is debatable, with some investigators arguing that mtROS are indispensable for inflammasome activation and others suggesting that mtROS only partially contribute to inflammasome activity (Munoz-Planillo et al., 2013; Harijith et al., 2014; Gross et al., 2016). Of interest, activation of the transcription factor nuclear factor erythroid 2-related factor 2 (Nrf2), which mediates transcription of antioxidant genes, has been shown to inhibit inflammasome-driven IL-1β maturation, supporting a clear role for total cellular ROS in promoting inflammasome assembly (Tsai et al., 2011; Ka et al., 2015; Hennig et al., 2018). It is currently unclear how much of this response is driven by mtROS specifically; however, it is reasonable to expect mtROS play a fairly large role because mitochondria are major drivers of ROS production (Brieger et al., 2012). Evidence suggests mtROS can further disrupt lysosomal compartments (Heid et al., 2013). On the other hand, lysosomal leakage has been previously shown to occur upstream from perturbations in mitochondrial membrane potential following cSiO_2_ exposure in AMΦs (Thibodeau et al., 2004; Joshi and Knecht, 2013). Taken together, these findings suggest that lysosomal and mitochondrial dysfunction might reciprocally influence one another in the context of particle-induced toxicity. Such a notion requires additional study, as the mechanisms driving cyclical lysosomal and mitochondrial dysfunction remain unclear.

#### Endoplasmic Reticulum Stress

Similar to mitochondrial dysfunction, ER stress has also been linked to inflammasome activation. Extracellular ATP, a mtDAMP released from dying cells, stimulates the transcription factor CCAAT enhancer binding protein homologous protein (CHOP) in LPS-primed BMDMΦs to induce Ca^2+^ signaling, which promotes Ca^2+^ efflux from the ER, downstream mitochondrial damage, and resultant caspase-1 activation (Murakami et al., 2012). Additionally, ER stress promotes NF-κB-dependent transcription of pro-IL-1β and activation of the oxidative protein folding pathway to induce ROS production (Kim et al., 2014). Elevated ROS levels initiate the dissociation of thioredoxin-interacting protein (TXNIP) from thioredoxin (TXN) and its subsequent association with the LRR of NLRP3, which promotes inflammasome oligomerization and caspase-1 activation (Kim et al., 2014). Furthermore, ER stress can activate inositol-requiring enzyme 1 alpha (IRE1α), which promotes translocation of TXNIP to the mitochondria and the release of mtDAMPs including mtROS and mtDNA (Zhou et al., 2020). In previous studies, it has been demonstrated in macrophages that SiO_2_ upregulates CHOP (Chen et al., 2019), asbestos increases CHOP expression and cytosolic Ca^2+^ (Ryan et al., 2014), and MWCNTs promote intracellular lipid accumulation, CHOP phosphorylation, and CD36 expression (Long et al., 2019). Additional research is needed to determine the specific steps that occur between particle phagocytosis and downstream ER stress.

Taken together, inflammasome-activating exogenous (Table 2) and endogenous (Table 3) particles have multifaceted impacts on intracellular lysosomal, mitochondrial, and ER-related functionality, and these pathways can feed into each other to mount robust inflammatory responses that drive rheumatic and autoimmune disease.

**TABLE 2:** Examples of studies demonstrating toxic responses of exogenous particles.

Reference	Particle Type(s)	Experimental Model(s)	Dose(s)	Time Point(s)	Results
Dostert et al. (2008)	SiO_2_, Asbestos	THP-1 cells (human MΦs)	SiO_2_: 0.2 mg/ml Asbestos: 0.2 mg/ml	6 h	Caspase-1 activation, IL-1β release
Hornung et al. (2008)	SiO_2_, Alum	Primary murine BMDMΦs, primary human PBMCs	SiO_2_: 125–1,000 µg/ml	3 h	LMP, cathepsin B release, caspase-1 activation, IL-1β release
			Alum: 100–500 µg/ml		
Kool et al. (2008)	Alum	Primary murine peritoneal MΦs	40–240 µg/ml	6 h	Caspase-1 activation, IL-1β maturation
Hamilton et al. (2009)	TiO_2_	Primary murine AMΦs	50–200 µg/ml	1 h, 4 h	LMP, cathepsin B release, ROS production, IL-1β release
Winter et al. (2011)	SiO_2_, TiO_2_	Primary murine BMDCs	SiO_2_: 5–50 µg/cm^2^	18 h	SiO_2_: apoptosis; TiO_2_: ROS production, IL-1β release
			TiO_2_: 5–50 µg/cm^2^		
Palomaki et al. (2011)	Asbestos, CNTs	Primary human MΦs	Asbestos: 100 µg/ml	6 h	Cathepsin B activity, Syk activity, ROS production, IL-1β release
			CNTs: 100 µg/ml		
Kuroda et al. (2011)	Alum	Primary murine peritoneal MΦs, primary murine BMDMΦs	400 µg/ml	2 h, 6 h	LMP, IL-1β synthesis, PGE_2_ synthesis
Joshi and Knecht (2013)	SiO_2_	MH-S AMΦs (murine AMΦs)	50 µg/cm^2^	30–120 min, 3–6 h	30–120 min: LMP; 3–6 h: caspase-3/9 activation, apoptosis, necrosis
Khameneh et al. (2017)	SiO_2_, Alum	Primary murine BMDCs	SiO_2_: 62.5–250 µg/ml	24 h	Syk activity, IL-2 release, CD4^+^ T cell expansion
			Alum: 62.5–250 µg/ml		
Desai et al. (2017)	SiO_2_, Asbestos	Primary murine neutrophils, primary human neutrophils	SiO_2_: 0.2 mg/ml Asbestos: 0.2 mg/ml	2 h	NET formation, primary necrosis and necroptosis, NET release

Alum, aluminum-containing salts; AMΦ, alveolar macrophage; BMDC, bone marrow-derived dendritic cell; BMDMΦ, bone marrow-derived macrophage; CNT, carbon nanotube; h, hour(s); LMP, lysosomal membrane permeabilization; min, minute(s); MΦ, macrophage; NET, neutrophil extracellular trap; PBMC, peripheral blood mononuclear cell; PGE_2_: prostaglandin E_2_; ROS, reactive oxygen species; SiO_2_, silicon dioxide; Syk, spleen tyrosine kinase; TiO_2_, titanium dioxide.

**TABLE 3:** Examples of studies demonstrating toxic responses of endogenous particles.

Reference	Particle Type(s)	Experimental Model(s)	Dose(s)	Time Point(s)	Results
Martinon et al. (2006)	MSU, CaP	THP-1 cells (human MΦs), primary human monocytes, primary murine peritoneal MΦs	MSU: 1–100 µg/ml CaP: 1–100 µg/ml	6 h	Caspase-1 activation, IL-1β maturation and release
Duewell et al. (2010)	CCs	Primary human PBMCs	15.6–125 µg/ml	6 h	LMP, caspase-1 activation, IL-1β release
Rajamaki et al. (2010)	CCs	THP-1 cells (human MΦs), primary human monocytes, primary human BMDMΦs	0.1–2 mg/ml	4–24 h	LMP, cathepsin B release, K^+^ efflux, IL-1β release
Conforti-Andreoni et al. (2011)	MSU	Primary murine BMDCs	250 µg/ml	5 days	Inflammasome activity and Th17-associated cytokine release
Pazar et al. (2011)	CaP	THP-1 cells (human MΦs), primary human monocytes, primary human MΦs, primary murine BMDMΦs	500 µg/ml	6 h	ROS production, caspase-1 activation, IL-1β release, apoptosis
Mulay et al. (2013)	CaOx	Primary murine BMDCs	30–1,000 µg/ml	6 h	CaOx phagocytosis, K^+^ efflux, IL-1β maturation and release
Desai et al. (2017)	MSU, CCs, CaP, CaOx	Primary murine neutrophils, primary human neutrophils	MSU: 0.2 mg/ml CCs: 0.2 mg/ml CaP: 0.2 mg/ml CaOx: 0.2 mg/ml	2 h	NET formation, primary necrosis and necroptosis, NET release

BMDC, bone marrow-derived dendritic cell; BMDMΦ, bone marrow-derived macrophage; CaOx, calcium oxalate; CaP, calcium phosphate; CC, cholesterol crystal; d, day(s); h, hour(s); LMP, lysosomal membrane permeabilization; MSU, monosodium urate; MΦ, macrophage; NET, neutrophil extracellular trap; PBMC, peripheral blood mononuclear cell; ROS, reactive oxygen species.

## Particle-Induced Cell Death Pathways That Contribute to Innate and Adaptive Immune Responses

Consistent with Matzinger’s danger model (Gallucci and Matzinger, 2001), exposure to exogenous (Table 2) and endogenous (Table 3) particles can trigger inflammasome-dependent and -independent cell death pathways in phagocytes, resulting in the release of DAMPs and autoantigens that can activate innate and adaptive immunity. Of note for the present review are necrosis, pyroptosis, apoptosis, necroptosis, and NETosis. In addition, we provide a brief perspective on PANoptosis, a recently proposed unified cell death pathway involving pyroptosis, apoptosis, and necroptosis.

### Necrosis

**FIGURE 4:**
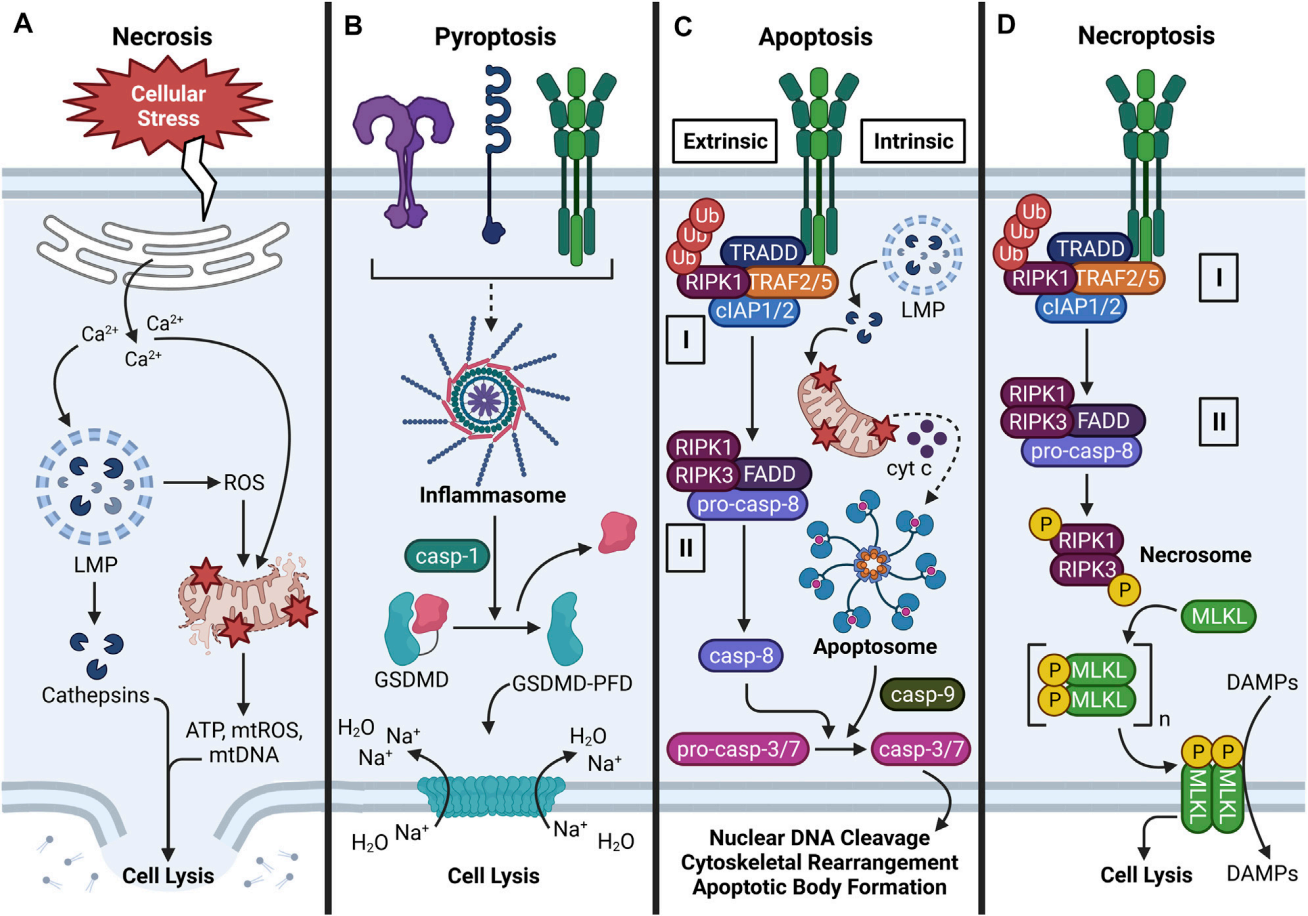
Major cell death pathways induced by particles. **(A)** Overview of necrosis. Necrosis can be triggered by various stimuli that provoke cellular stress. Common hallmarks of necrosis include Ca^2+^ efflux from the ER, Ca^2+^-induced LMP and cathepsin release, ROS-driven mitochondrial dysfunction, cellular swelling, plasma membrane rupture, and DAMP release. **(B)** Overview of pyroptosis. Following NLRP3 inflammasome oligomerization and activation, caspase-1 proteolytically processes GSDMD to expose its N-terminal pore-forming domain (PFD). GSDMD-PFD polymerizes into a pore in the plasma membrane, which allows Na^+^ to move along its electrochemical gradient into the cell. By osmosis, water enters the cell, causes cellular swelling, and cell lysis. **(C)** Overview of apoptosis (extrinsic and intrinsic). Extrinsic apoptosis is triggered by activation of a death receptor (e.g., TNFR), which promotes assembly of Complex I. Complex I consists of TRADD, TRAF2/5, cIAP1/2, and ubiquitinated RIPK1. Inhibition of cIAP1/2 and/or deubiquitylation of RIPK1 by CYLD (not shown) induces formation of cytosolic Complex II, which consists of RIPK1, RIPK3, FADD, and pro-caspase-8 oligomers that proteolytically activate themselves. Intrinsic apoptosis is defined by release of cytochrome *c* (cyt *c*) from perturbed mitochondria, formation of a multiprotein apoptosome, and activation of caspase-9. In certain cases, LMP-driven cathepsin release may contribute to mitochondrial dysfunction. Caspase-8/9 proteolytically activates caspase-3/7, which promotes nuclear DNA cleavage, cytoskeletal rearrangement, and apoptotic body formation. **(D)** Overview of necroptosis. Necroptosis is characterized by activation of a death receptor (e.g., TNFR), Complex I formation, and Complex II formation as in extrinsic apoptosis. Inhibition of pro-caspase-8 activation allows formation of the RIPK1-RIPK3 necrosome, which phosphorylates MLKL. Phospho-MLKL monomers polymerize into a pore-shaped complex at phosphatidylinositol 3-phosphate sites in the inner leaflet of the plasma membrane. Consequently, cell lysis occurs and DAMPs are released from the cell. Some steps in the depicted cell death pathways are omitted for clarity.

Necrosis is an unprogrammed cell death pathway characterized by organellar disorganization, cellular swelling, plasma membrane rupture, and DAMP release (Rello et al., 2005). No specific signaling pathway is associated with necrosis, but it is usually preceded by lysosomal rupture, mitochondrial swelling, and ROS production (Green and Llambi, 2015; Niemann and Rohrbach, 2016). Necrosis is generally considered a proinflammatory mode of cell death, as DAMPs released from dying cells provoke inflammatory gene expression and signaling in neighboring innate and adaptive immune cells (Davidovich et al., 2014) (Figure 4A). Both exogenous and endogenous particles have been shown to provoke necrotic cell death in a variety of cell types including AMΦs, fibroblasts, mesothelial cells, and kidney epithelial cells. Primary mechanisms by which particles induce necrosis include upstream LMP, mitochondrial depolarization, and ROS production (Joshi and Knecht, 2013; Mulay et al., 2019b; Ito et al., 2020), though it might also be possible that particles directly disrupt the plasma membrane (Pavan et al., 2020; Reus et al., 2020) or promote necrosis through other unelucidated mechanisms. Infectious agents, mechanical stress, hypoxia, and chemical and radiation exposure can also compromise the integrity of the cell membrane, leading to necrosis (Nirmala and Lopus, 2020). When particles induce necrosis, the dying cell releases particles and DAMPs, which can perpetuate unresolved inflammation if not efficiently cleared.

### Pyroptosis

Pyroptosis is a programmed lytic cell death pathway that is dependent on inflammasome activation (Lamkanfi and Dixit, 2014; Abe and Morrell, 2016). As previously discussed, many different types of exogenous and endogenous particles can activate the inflammasome (Martinon et al., 2006; Dostert et al., 2008; Hornung et al., 2008; Tsugita et al., 2017a; Dominguez-Gutierrez et al., 2018; Karasawa and Takahashi, 2019; Luz et al., 2019). When the NLRP3 inflammasome assembles and activates caspase-1 following particle exposure, caspase-1 not only converts pro-IL-1β and pro-IL-18 to their mature forms but also cleaves the N-terminal pore-forming domain (PFD) of gasdermin D (GSDMD). PFD monomers oligomerize and insert into the plasma membrane, which destabilizes plasma membrane potential and leads to an osmotic movement of water into the cell that mediates cellular swelling and lysis (Ros et al., 2020) (Figure 4B). Like necrosis, pyroptosis is considered a proinflammatory cell death pathway because the GSDMD pore and resultant lysis caused by its insertion into the plasma membrane allows passage of DAMPs from intracellular to extracellular environments (Davidovich et al., 2014).

### Apoptosis

Exposure to exogenous and exogenous particles such as SiO_2_, asbestos, CCs, MSU, and CaP can induce apoptosis in macrophages (Hamilton et al., 1996; Geng et al., 2003; Pazar et al., 2011; Joshi and Knecht, 2013; Kim et al., 2016). Unlike necrosis, apoptosis is morphologically defined by nuclear DNA cleavage, cytoskeletal rearrangement, cellular shrinkage, and plasma membrane blebbing (Rello et al., 2005) (Figure 4C). In apoptosis, the plasma membrane does not rupture but rather invaginates organelles and DAMPs in apoptotic bodies that are engulfed by phagocytes (Santavanond et al., 2021). Accordingly, apoptosis is a quiescent mode of cell death; however, if apoptotic bodies are insufficiently removed, they undergo secondary necrosis, which releases DAMPs into the extracellular space (Nagata, 2018). Apoptosis can be induced by death receptor (DR) signaling (extrinsic pathway), mitochondrial signaling (intrinsic pathway), or perforin/granzyme signaling (Elmore, 2007; Nirmala and Lopus, 2020). The perforin/granzyme pathway falls outside the scope of the present review, but readers are encouraged to consult other excellent reviews on this topic (Trapani and Smyth, 2002; Voskoboinik et al., 2010; Voskoboinik et al., 2015). While particles have not been shown to bind DRs and particle-sensing receptors are not known to activate signaling components downstream from DRs (Thibodeau et al., 2003; Hu et al., 2006), an overview of extrinsic apoptosis is warranted because particle exposure can induce expression and secretion of DR ligands such as TNF-α (Dubois et al., 1989; Perkins et al., 1993; Gozal et al., 2002; Brown et al., 2007; Chen et al., 2018; Dominguez-Gutierrez et al., 2018). In the context of particle-triggered apoptosis, however, the intrinsic pathway is most relevant because particles can indirectly elicit mitochondrial stress (Thibodeau et al., 2003; Hu et al., 2006; Joshi and Knecht, 2013).

In the extrinsic pathway, the initiation phase is triggered by activation of a DR in the TNF receptor superfamily [e.g., TNF receptor (TNFR)-1 or Fas receptor (FasR)] by its corresponding ligand [e.g., TNF-α or Fas ligand (FasL)], which triggers association of an adapter protein to the intracellular domain of the DR (Nirmala and Lopus, 2020). The recruited adapter protein differs depending on the DR activated: FasL recruits Fas-associated protein with death domain (FADD) to FasR, and TNF-α recruits TNFR1-associated death domain protein (TRADD) to TNFR1 (Elmore, 2007). Specific to TNFR1, TNF receptor associated factor (TRAF)-2/5, receptor-interacting serine/threonine-protein kinase (RIPK)-1, and cellular inhibitor of apoptosis protein (cIAP)-1/2 are subsequently recruited to the intracellular receptor domain of TNFR1 and associate with TRADD (i.e., Complex I). Cylindromatosis tumor suppressor protein (CYLD) then deubiquitylates RIPK1 which allows this protein to leave Complex I and leads to association of FADD and RIPK3 (i.e., Complex II). Following these events, FADD associates with multiple pro-caspase-8 proteins to form a death-inducing signaling complex (DISC) that cleaves pro-caspase-8 to caspase-8 (Tummers and Green, 2017). Caspase-8 then proteolytically activates caspase-3 and -7 and triggers the execution phase of apoptosis (Elmore, 2007). During the execution phase, mature caspases-3 and -7 cleave nuclear DNA and intracellular proteins, which are encapsulated in apoptotic bodies (Walsh et al., 2008). Apoptotic cells express phosphatidylserine (PS) in the outer leaflet of the plasma membrane, which serves as an “eat me” signal for phagocytes to engulf the dying cells. This process, termed efferocytosis, functions to remove apoptotic bodies, thus preventing secondary necrosis and DAMP release (Segawa and Nagata, 2015).

In the intrinsic pathway, particle-driven organellar dysfunction leads to MPTP opening, as described in the previous section. This releases cytochrome *c* (cyt *c*) into the cytosol, where it binds with apoptotic protease activating factor 1 (Apaf-1) and pro-caspase-9 to form a multiprotein apoptosome complex that is structurally and functionally analogous to the inflammasome. During this process, mitochondrial second mitochondria-derived activator of caspases (SMAC) and high temperature requirement protein A2 (HtrA2) block the activity of inhibitors of apoptosis proteins (IAPs) to promote apoptosis (D’Arcy, 2019). Pro-caspase-9 moieties proteolytically activate each other within the apoptosome in a manner that resembles caspase-1 activation in the inflammasome. Activated caspase-9 then activates caspase-3, which activates caspase-activated DNase (CAD). Subsequently, caspase-3 cleaves nuclear DNA, triggers cytoskeletal rearrangement, and induces formation of apoptotic bodies, which are cleared by phagocytes under normal conditions (Elmore, 2007). However, phagocytotic capacity might be exhausted under conditions of persistent particle exposure, which raises the likelihood of secondary necrosis, DAMP release, and ongoing inflammatory signaling.

### Necroptosis

Exogenous particles (e.g., SiO_2_, TiO_2_) and endogenous particles (e.g., CCs, MSU, CaP, CaOx) have been demonstrated to induce necroptosis in neutrophils, with less well-defined effects in macrophages (Desai et al., 2017; Honarpisheh et al., 2017). Necroptosis is a programmed cell death pathway that morphologically resembles necrosis but shares cellular machinery with the extrinsic apoptotic pathway. Accordingly, the early steps of necroptosis involve DR activation and recruitment of signaling proteins to the intracellular domain of the DR (e.g., TNFR1) to form Complex I as previously described (Frank and Vince, 2019). TNFR1 endocytosis, cIAP1/2 inhibition, and RIPK1 deubiquitylation by CYLD triggers formation of cytosolic Complex II, which involves dissociation of TRAF2/5 and cIAP1/2 and association of FADD and pro-caspase-8 as previously described (Newton and Manning, 2016; Su et al., 2016). Under normal conditions, Complex II can induce extrinsic apoptosis. However, impairment of pro-caspase-8 activity allows formation of a RIPK1- and RIPK3-containing complex called the necrosome (Galluzzi et al., 2017). The necrosome facilitates activation of the pseudokinase mixed lineage kinase domain-like (MLKL) via phosphorylation, and MLKL monomers forms oligomers at phosphatidylinositol 3-phosphate sites on the inner leaflet of the plasma membrane. Consequently, the MLKL oligomers elicit plasma membrane permeabilization by currently undefined mechanisms, leading to destabilization of membrane potential and cell lysis (Figure 4D). As with necrosis and pyroptosis, necroptosis allows DAMP release from the cell, and these DAMPs can induce downstream inflammatory responses (Newton and Manning, 2016). While the exact mechanisms of particle-induced necroptosis have yet to be fully elucidated, it is possible that cathepsins released from disrupted phagolysosomes promote assembly of the RIPK1-RIPK3 necrosome, which promotes MLKL polymerization (Honarpisheh et al., 2017). Another possibility is that TNF-α released from dying cells interacts with TNFR1 on viable nearby cells, promoting either extrinsic apoptosis or necroptosis depending on pro-caspase-8 activity.

### PANoptosis

**FIGURE 5:**
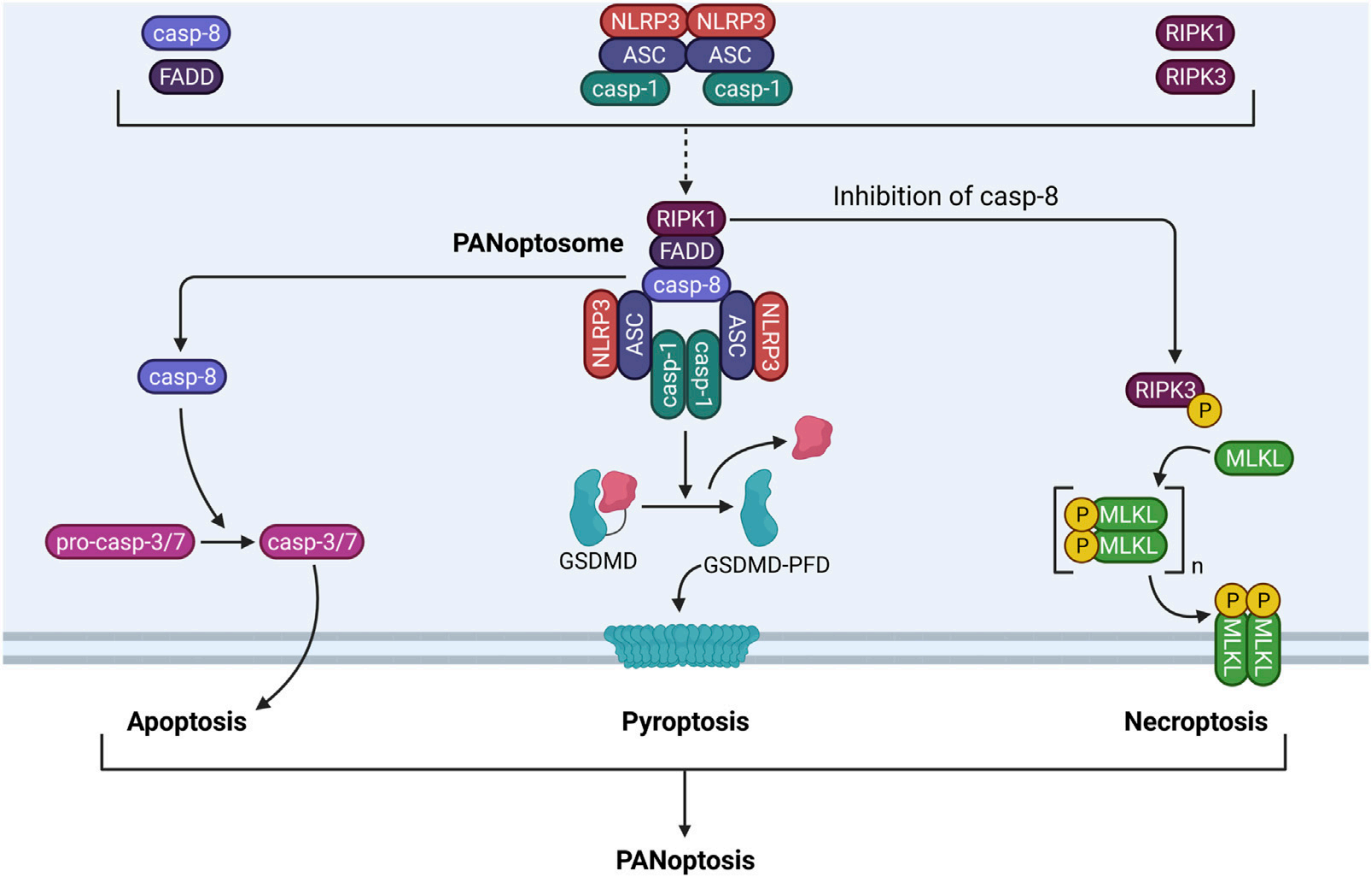
PANoptosome components and functionality. The PANoptosome is a multiprotein complex consisting of molecules from the apoptotic, pyroptotic, and necroptotic cell death pathways. Exposure to a proinflammatory stimulus such as LPS causes upregulation and activation of apoptotic proteins (i.e., caspase-8, FADD), pyroptotic proteins (i.e., NLRP3, ASC, caspase-1), and necroptotic proteins (i.e., RIPK1, RIPK3). These proteins associate with one another to form the PANoptosome. Following assembly, the PANoptosome can execute apoptosis, pyroptosis, and necroptosis simultaneously by driving caspase-3/7 activation by caspase-8, GSDMD processing by caspase-1, and MLKL phosphorylation and pore formation by RIPK1 and RIPK3. Cell death by concurrent apoptosis, pyroptosis, and necroptosis is termed PANoptosis.

PANoptosis is a recently coined term that unifies inflammatory cell death involving simultaneous activation of pyroptosis, apoptosis, and necroptosis (Malireddi et al., 2019). Currently, two models have been proposed for PANoptosis-induced cell death. In the first model, an inflammatory stimulus simultaneously activates the inflammasome, apoptosome, and necrosome, which execute their respective forms of cell death. In the second model, PANoptosis is induced through inflammatory stimuli that trigger formation of a multiprotein complex called the PANoptosome, which triggers pyroptosis, apoptosis, and necroptosis at the same time. In myeloid-lineage phagocytes (e.g., neutrophils and macrophages exposed to LPS), caspase-8 (apoptosis), FADD (apoptosis and necroptosis), RIPK1 (necroptosis), NLRP3 (pyroptosis), ASC (pyroptosis), and caspase-1 (pyroptosis) can assemble into the PANoptosome. Accordingly, the PANoptosome can trigger apoptosis by caspase-8-dependent activation of caspase-3/7, pyroptosis by caspase-1-dependent cleavage of GSDMD, and necroptosis by RIPK3-dependent phosphorylation of MLKL (Samir et al., 2020) (Figure 5). The result is a detrimental cell death pathway that permits release of inflammatory DAMPs into the extracellular space. While it is still unclear which factors dictate execution of PANoptosis versus individual activation of pyroptosis, apoptosis, or necroptosis, inhibition of TGF-β-activated kinase 1 (TAK1) has previously been associated with PANoptosome formation in macrophages (Malireddi et al., 2020).

Currently, there is no evidence linking particle exposure to PANoptosis in myeloid-lineage phagocytes, yet the current evidence supports such a possibility. Multiple particles have been previously reported to induce pyroptosis, apoptosis, and necroptosis in phagocytes [summarized by Mulay et al. (2019a)], but whether these multiple forms of cell death occur simultaneously in the same model is yet to be determined. Intriguingly, components of these three pathways can regulate one another. Not only can caspase-8 promote pyroptosis by cleaving GSDMD, but it can also prevent necroptosis by degrading RIPK. Necroptotic MLKL pore formation also can trigger NLRP3 inflammasome activity by K^+^ efflux (Schwarzer et al., 2020).

### NETosis

**FIGURE 6:**
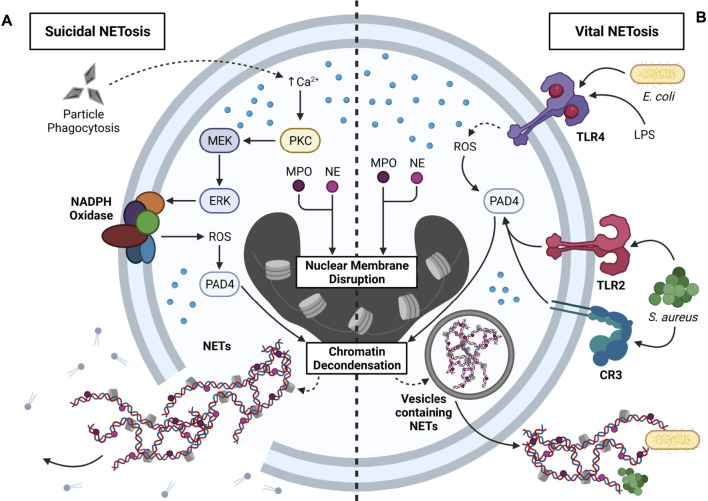
Mechanisms that contribute to NETosis. NETosis is the process by which neutrophil extracellular traps (NETs) are formed and released from neutrophils. Two primary forms of NETosis exist: suicidal and vital NETosis. **(A)** Overview of suicidal NETosis. Neutrophils phagocytose exogenous particles (e.g., SiO_2_) and endogenous particles (e.g., CCs), which triggers Ca^2+^ efflux from the ER. Intracellular Ca^2+^ efflux activates protein kinase C (PKC), PKC activates MEK, and MEK activates ERK. ERK stimulates NADPH oxidase via gp91phox phosphorylation, and NADPH oxidase produces ROS. ROS activates peptidyl arginine deiminase 4 (PAD4), which contributes to chromatin decondensation. Translocation of myeloperoxidase (MPO) and neutrophil elastase (NE) into the nucleus leads to nuclear membrane disruption and additional chromatin decondensation. Resultant NETs are directly released into the cytosol, and rupture of the plasma membrane contributes to extracellular NET release and neutrophil death. Suicidal NETosis occurs within a 2–4 h timeframe. **(B)** Overview of vital NETosis. Activation of TLR4 by LPS or Gram-negative bacteria (e.g., *E. coli*) contributes to ROS production, which is required for PAD4 activity. Alternatively, activation of TLR2 or CR3 by Gram-positive bacteria (e.g., *S. aureus*) leads to downstream PAD4 activation. As with suicidal NETosis, PAD4 triggers chromatin decondensation, and nuclear translocation of MPO and NE contributes to disruptions in the nuclear membrane. NETs are encased in nuclear vesicles, and NETs are released from viable neutrophils via exocytosis. Vital NETosis occurs within a 5–60 min timeframe, and released NETs can ensnare bacteria in the extracellular environment.

In addition to pyroptosis, apoptosis, and necroptosis, exogenous particles (e.g., SiO_2_, alum) and endogenous particles (e.g., MSU, CCs, CaP) can induce NETosis [reviewed by (Rada, 2017) and (Li et al., 2018)]. NETosis describes the process by which neutrophil extracellular traps (NETs) are formed within and released from neutrophils (Sollberger et al., 2018). NETs are web-like structures composed of decondensed chromatin decorated with cytosolic myeloperoxidase (MPO) and neutrophil elastase (NE) (Papayannopoulos, 2018). NETs can be released from neutrophils by two mutually exclusive pathways: 1) suicidal NETosis and 2) vital NETosis (Yipp and Kubes, 2013) (Figure 6).

In suicidal NETosis, phagocytosis of particles elicits Ca^2+^ efflux from the ER, which triggers activation of protein kinase C (PKC). PKC activates the MEK/ERK pathway, ERK phosphorylates the gp91phox subunit of NADPH oxidase to induce ROS production, and increased cytosolic ROS activate peptidyl arginine deiminase 4 (PAD4). Together with MPO and NE, which translocate to the nucleus, PAD4 promotes chromatin decondensation and nuclear membrane disruption. Consequently, NETs are released from the nucleus into the cytosol, where they are further decorated with cytosolic proteins, and ultimately released into the extracellular environment upon cell lysis (Delgado-Rizo et al., 2017; Jorch and Kubes, 2017). Unlike suicidal NETosis, in vital NETosis, NETs are packaged into vesicles and released by exocytosis, and thus, the neutrophil remains viable. Stimulation of TLR2/4 or CR3 by Gram-positive bacteria (e.g., *S. aureus*) or Gram-negative bacteria (e.g., *E. coli*) activates PAD4, which partners with nuclear MPO and NE to induce nuclear membrane disruption and chromatin decondensation. (Delgado-Rizo et al., 2017; Jorch and Kubes, 2017). While released NETs can immobilize bacteria and viruses, they can also potentiate inflammation (Papayannopoulos, 2018). This raises a few questions pertaining to NETosis and particle toxicology. First, can released NETs capture extracellular particles and prevent their interactions with other phagocytes? Second, can NETs in particle-exposed neutrophils be decorated with particles prior to their release? Answering these questions could provide further insight into the protective and/or pathologic roles of NETs in particle-driven diseases.

## Physicochemical Attributes That Influence Particle-Induced Toxicity and Proinflammatory Responses

Although many published studies suggest that different particles elicit similar toxic mechanisms in myeloid-lineage phagocytes, these responses depend greatly on the physicochemical attributes of the particle. Such attributes may include, but are not limited to, particle length (Hamilton et al., 2009; Murphy et al., 2011; Boyles et al., 2015), size (Fenoglio et al., 2012; Kusaka et al., 2014; Mischler et al., 2016), shape (Turci et al., 2016; Cohignac et al., 2018; Nahle et al., 2019), surface area (Sager and Castranova, 2009; Rabolli et al., 2010), and surface charge (Morishige et al., 2010; Hamilton et al., 2012; Hamilton et al., 2018; Pavan et al., 2020). An in-depth discussion of these attributes goes beyond the scope of this review, but the reader is encouraged to consult other previously published reviews on this topic (Aust et al., 2011; Rabolli et al., 2016; Sukhanova et al., 2018; Baranov et al., 2020). While current research has focused on characterizing relationships between particle attributes and toxic responses exhibited by exogenous particles, these relationships have not yet been characterized in relation to endogenous particles.

## From Particle Exposure to Loss of Immunological Self-Tolerance

As discussed in previous sections, inflammasome activity and cell death induced by exogenous particles (Table 2) and endogenous particles (Table 3) permit DAMP release into the extracellular environment, where they can stimulate innate and adaptive immune cells. Released DAMPs include proinflammatory cytokines, nucleic acids, uric acid, cholesterol, heat shock proteins, HMGB1, type I interferons (IFNs), NETs, and mtDAMPs including mtDNA, ATP, cardiolipin, and cyt *c* [reviewed by Gallo and Gallucci (2013) and Grazioli and Pugin (2018)]. DCs, which are commonly referenced as bridges between innate and adaptive immunity (Balan et al., 2019), may also be bridges between particle exposure and loss of immunological self-tolerance because they interact with both particles and released DAMPs (Gallo and Gallucci, 2013). For example, DCs secrete cytokines involved in Th1 and Th17 differentiation (i.e., IL-1α, IL-1β, IL-2, IL-6, IL-17, IL-23) in response to MSU (Conforti-Andreoni et al., 2011), CCs (Westerterp et al., 2017), or alum (Khameneh et al., 2017). SiO_2_ and TiO_2_ induce caspase-1-dependent IL-1β maturation and apoptotic cell death in DCs (Winter et al., 2011), and extracellular IL-1β plays critical roles in promoting Th17 polarization (Sutton et al., 2006). In addition, HMGB1, ATP, TNF-α, and NETs can stimulate DC maturation, proinflammatory cytokine production (i.e., IL-6, CXCL8, IL-12, TNF-α), and subsequent T cell activation (Schnurr et al., 2000; Yang et al., 2007; Parackova et al., 2020). Furthermore, specific DC subsets secrete type I IFN and B-cell activating factor (BAFF), which regulate B cell differentiation into antibody-secreting plasma cells (Jego et al., 2005). Intriguingly, DCs also can promote and maintain immunological tolerance by inducing regulatory T cell (Treg) differentiation through cell-to-cell contact or secreted cytokines such as TGF-β and IL-10 (Raker et al., 2015; Hilpert et al., 2019). Consequently, activated Tregs can suppress proliferation and differentiation of naïve T cells into effector T cells, as well as the functions of activated CD4^+^ and CD8^+^ T cells, B cells, macrophages, and DCs. Treg depletion has been associated with exacerbated immune responses to self- and non-self antigens and development of autoimmunity (Sakaguchi et al., 2008; Ma, 2020). Nonetheless, the impacts of Treg function on particle-driven inflammation remain unclear. For instance, imbalances in the Treg/Th17 ratio significantly aggravate SiO_2_- and MSU-induced inflammation in the lungs and joints of mice, respectively (Dai et al., 2016; Dai et al., 2018), but inhaled SiO_2_ and asbestos elicit recruitment of Tregs to the lungs, which secrete TGF-β and IL-10 and contribute to resultant development of pulmonary fibrosis (Liu et al., 2010; Lo Re et al., 2011; Maeda et al., 2017). Accordingly, DCs play crucial roles in regulating T cell differentiation, interacting with proximal particles and DAMPs, and maintaining immunological self-tolerance. Dysregulated DC activation by particles and DAMPs, on the other hand, represents one major bridge connecting particle-induced innate immunity to irregular adaptive immunity.

Cells undergoing particle-induced death not only release DAMPs into the extracellular space but also autoantigens that can be recognized by T and B cells and consequently trigger autoimmunity. Autoantigens are self-proteins that are erroneously recognized as foreign proteins by the host’s immune system (Burbelo et al., 2021). When presented by DCs or other antigen-presenting cells, autoantigens promote activation of autoreactive T cells, which evade elimination in individuals with genetic predispositions to autoimmune disease and specifically target the presented self-proteins (Stranges et al., 2007). In addition, autoreactive T cells promote differentiation of autoreactive B cells into plasma cells, which secrete autoantibodies specific to the presented self-proteins (Riedhammer and Weissert, 2015). Autoantigens involved in systemic autoimmune diseases such as systemic lupus erythematosus (SLE) include dsDNA, small nuclear ribonucleoprotein (snRNP), cardiolipin, and histone proteins (i.e., H2B, H3, H4) (Doyle et al., 2014; Rosen and Casciola-Rosen, 2016). In some cases, autoantigens with post-translational modifications (PTMs), but not native self-proteins, are recognized by autoreactive T and B cells (Doyle et al., 2014). These PTMs include phosphorylation/dephosphorylation (Terzoglou et al., 2006; Nagai et al., 2012), methylation (Brahms et al., 2000), acetylation (van Bavel et al., 2009), citrullination (Lande et al., 2021), oxidation (Chang et al., 2004), and isomerization (Doyle et al., 2013). Since cytotoxic processes can contribute to modification of autoantigen structure and immunogenicity, it is tempting to speculate that intracellular mechanisms involved in inflammasome activation may also contribute to formation of PTM autoantigens and novel autoantigens. For example, cathepsins released from particle-containing phagolysosomes may non-specifically cleave mitochondrial and cytosolic proteins to create novel self-proteins that elicit immunological autoreactivity when released from dying cells. Caspase-1 may cleave mitochondrial and cytosolic proteins other than its identified substrates (i.e., pro-IL-1β, pro-IL-18, GSDMD) at specific sites, though this possibility seems less likely.

In addition to the roles that released DAMPs, autoantigens, and other danger signals play in aberrant activation of the immune system, genetics constitute a major determinant in the loss of immunological self-tolerance and resultant development of autoimmunity. Although some autoimmune diseases are monogenic, the majority are polygenic by nature (Doria et al., 2012). Genetic polymorphisms leading to increased expression and activation of inflammasome proteins (e.g., NLRP3), TLRs (e.g., TLR7, TLR9), transcription factors (e.g., STAT4), and IFN signaling proteins (e.g., IRF5) have been associated with increased susceptibility and severity of several autoimmune diseases including SLE, rheumatoid arthritis (RA), and multiple sclerosis (Cho and Gregersen, 2011; Yang and Chiang, 2015). In addition, loss-of-function mutations in efferocytosis receptors (e.g., MerTK), which leads to decreased engulfment of cytotoxic cell debris, have been associated with systemic autoimmunity (Lemke, 2013). Unique to autoimmune diseases are genetic polymorphisms in the major histocompatibility complex (MHC), or human leukocyte antigen (HLA) region in humans (Caso et al., 2018), which is crucial for presenting antigens to CD4^+^ helper T cells (Gough and Simmonds, 2007). Taken together, these genetic aberrations set the stage for increased inflammasome priming and activation, elevated proinflammatory cytokine and IFN production, and hindered cell debris clearance contributing to inflammatory tissue damage. In individuals susceptible to autoimmunity, these genetic variants may also contribute to enhanced autoantigen presentation to T and B cells, tissue damage by autoreactive T cells, and autoantibody production by autoreactive plasma cells, leading to development of autoimmunity.

## Particle-Triggered Autoimmune and Autoinflammatory Diseases

Consistent with evoking inflammatory responses and cell death in phagocytes, exogenous and endogenous particles can trigger development of both chronic inflammatory and autoimmune diseases. Workplace inhalation of asbestos fibers has a long-recorded history of potentiating asbestosis and malignant mesothelioma (Westerfield, 1992; Brody, 1993; Bartrip, 2004). In rodents, CNT inhalation has been associated with proinflammatory AMΦ polarization and pulmonary fibrosis (Dong and Ma, 2018), (Kobayashi et al., 2017). TiO_2_ exposure has been connected to malabsorption, neuroinflammation, and cardiopulmonary inflammation in rodents and humans (Czajka et al., 2015; Zhao et al., 2018; Baranowska-Wojcik et al., 2020). MSU deposition in joints and blood vessels can promote gouty arthritis (Rock et al., 2013), coronary heart disease, and neurodegeneration (Jin et al., 2012). CCs can contribute to coronary heart disease (Goldstein and Brown, 2015), atherosclerosis (Nidorf et al., 2020), NASH (Ioannou, 2016), and cholesterol gallstone disease (Di Ciaula et al., 2018) if deposited in blood vessels, liver, or gallbladder, respectively. Furthermore, CaP and CaOx deposition can lead to pseudogout, nephropathy, and atherosclerosis (Lorenz et al., 2013; Kalampogias et al., 2016; Rosenthal and Ryan, 2016). Although different particles share similar mechanisms of promoting persistent inflammation, they elicit different pathologies depending on their routes of exposure and distribution in the body.

In addition to genetic predispositions, other factors that may modulate autoimmune susceptibility include particle exposure level, aging, and biological sex. Dose-response impacts of particle exposure on autoimmune pathogenesis remain largely uninvestigated. However, according to Paracelsus’s paradigm statement “The dose makes the poison,” it can be assumed that chronic exposures to many particles are more likely to induce aberrant inflammation and autoimmunity compared to acute exposures to few particles (Lison et al., 2014). This trend has been noted with respirable cSiO_2_ exposure in both mice (Bates et al., 2015; Mayeux et al., 2018) and humans (De Klerk et al., 2002; Boudigaard et al., 2021). Conversely, aging seems to have unclear impacts on the development of autoimmune disease. Older adults (>60 years) have higher prevalence of non-organ-specific autoantibodies than younger adults (20–60 years), but older adults are less likely than younger adults to develop autoimmune disease (Vadasz et al., 2013). Accordingly, aging contributes to restructuring of the immune system, leading to impaired immune responses, increased inflammation and oxidative stress, and increased autoantibody production (Watad et al., 2017). This suggests that the immune system is much more sensitive and reactive to autoantigens in younger adults compared to older adults, as many systemic autoimmune diseases manifest between 30 and 50 years of age (Amador-Patarroyo et al., 2012). A third factor that influences autoimmunity yet remains an enigma is biological sex. In general, autoimmune disease is more prevalent in women compared to men (Ngo et al., 2014). Postulated reasons for this observation include pregnancy and hormonal changes during puberty and menopause (Angum et al., 2020). While particle-induced inflammation and autoimmunity might be more biased toward men working in dusty occupations, more women are beginning to enter similar occupations, with emphases on making dental molds and using scouring powders in custodial work (Finckh et al., 2006; Pollard, 2012).

While exposure to exogenous and endogenous particles has been linked to inflammatory and autoimmune diseases, much less is known about their roles in initiating and exacerbating autoinflammatory disease. Briefly, autoinflammatory diseases are defined by uncontrolled innate immunity contributing to direct tissue damage and disease pathogenesis, whereas autoimmune diseases are potentiated by unresolved innate immunity leading to hyperactivation of adaptive immunity, the latter of which primarily drives tissue damage and disease pathogenesis (Doria et al., 2012). Most autoinflammatory diseases are caused by genetic mutations contributing to aberrant inflammasome activity, IL-1β activation, protein folding, IFN signaling, complement activation, and proinflammatory cytokine signaling (Krainer et al., 2020). Considering these mechanisms, it is not unreasonable to speculate that particles can worsen, or even trigger, autoinflammatory disease, beginning with myeloid-lineage phagocytes. Research in this area is crucial for verifying an etiological link between particle exposure and autoinflammatory disease and would provide additional rationale for regulating workplace particle exposure and fine-tuning dietary constituents for individuals predisposed to either autoinflammatory or autoimmune disease.

## Linking Particle-Induced Inflammation to Autoimmune disease—Crystalline Silica as a Prototypical Example

**FIGURE 7:**
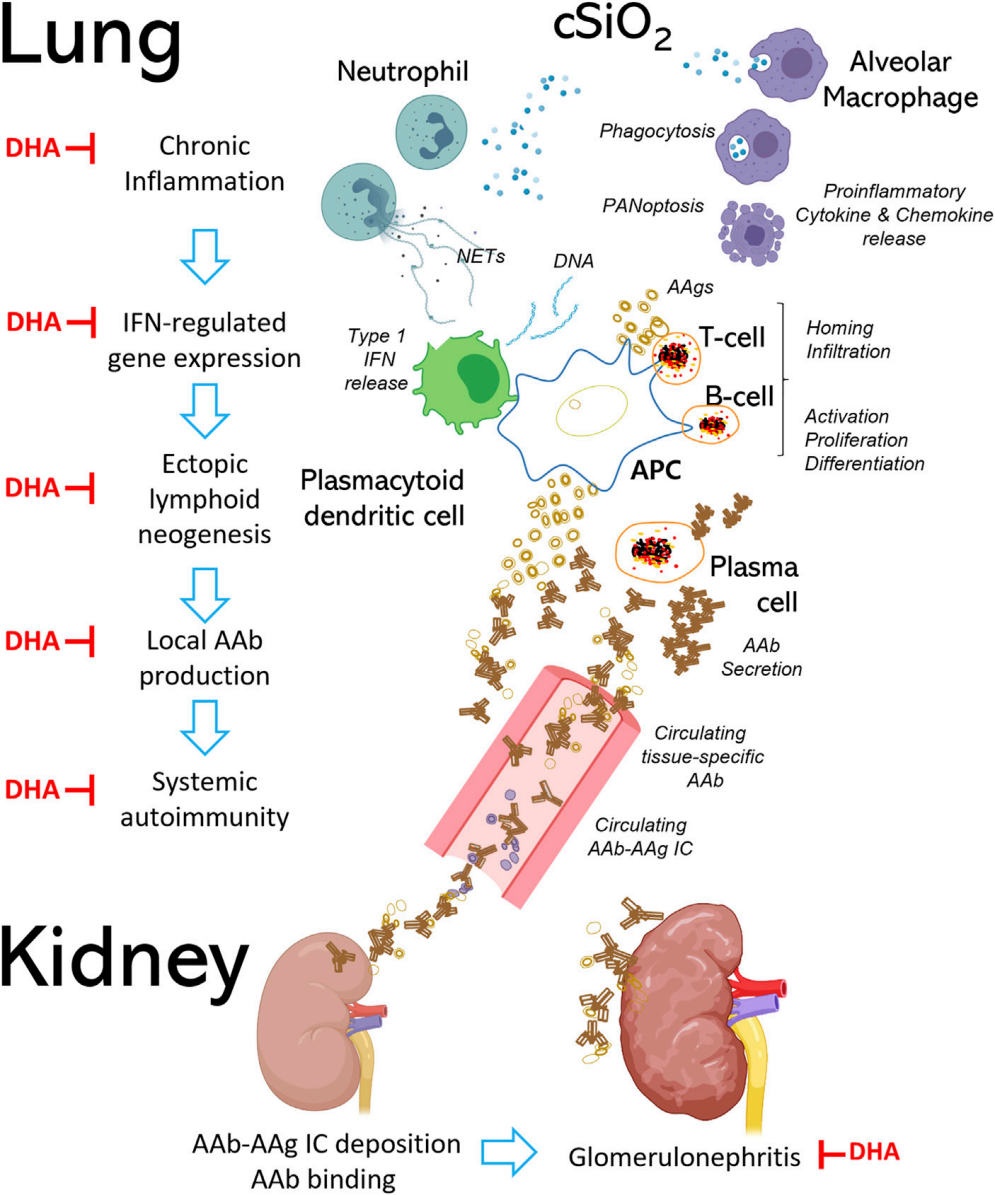
Respirable cSiO_2_ triggers autoimmune flaring and progression in the SLE-prone female NZBWF1 mouse. Chronic exposure to respirable cSiO_2_ particles contributes to irresolvable lung inflammation and systemic autoimmunity, resulting in end-stage glomerulonephritis and shortened lifespan in female NZBWF1 mice. Alveolar macrophages (AMΦs), which serve as one of the first lines of immunological defense in the lung, detect and phagocytose inhaled cSiO_2_. Resultantly, cSiO_2_ particles engulfed by AMΦs induce immunogenic cell death (i.e., pyroptosis, apoptosis, necrosis), proinflammatory cytokine and chemokine release, and NETosis in neighboring neutrophils. Aberrant accumulation of dead cell corpses, proinflammatory mediators, and host nucleic acids promotes recruitment of autoreactive T and B cells into the lung and type I interferon (IFN) release from plasmacytoid dendritic cells, leading to formation of ectopic lymphoid tissue (ELT). Type I IFN triggers maturation of B cells into plasma cells, which secrete IgG autoantibodies (AAb) that target local and systemic autoantigens (AAg). Binding of AAbs to their corresponding AAgs can lead to formation of immune complexes (ICs) that circulate in the body via blood vessels and deposit in other organs such as the kidneys. Once deposited, ICs recruit additional proinflammatory cells to the tissue, ultimately resulting in irreversible kidney damage and failure. Steps at which DHA has been shown to interfere with these pathways are indicated by red ┴ symbols.

Both preclinical and clinical studies have established that exposure to respirable cSiO_2_ contributes to SLE and other human autoimmune diseases (Parks et al., 2002; Pollard, 2016; Morotti et al., 2021). Patients with SLE typically have recurrent cycles of flaring and remission that eventually can cause cumulative damage to kidney, lung, heart, skin, and/or brain (Moulton et al., 2017). Intriguingly, both autoimmune flaring and disease progression can be induced by instilling cSiO_2_ to airways of mouse models of SLE (Brown et al., 2003; Brown et al., 2004; Brown et al., 2005; Bates et al., 2015; Clark et al., 2017; Bates et al., 2018; Foster et al., 2019). This is perhaps best exemplified in SLE-prone female New Zealand Black White (F1) (NZBWF1) mice which show autoantibody-driven glomerulonephritis with proteinuria by age 34 weeks resulting in death by age 52 weeks (Borchers et al., 2000). Our laboratory has demonstrated in this model that four weekly intranasal cSiO_2_ instillations of 1 mg trigger glomerulonephritis 12 weeks earlier than the conventional genome-driven model (Bates et al., 2015; Bates et al., 2018). Before glomerulonephritis onset in these mice*,* cSiO_2_ elicits severe pulmonary pathology involving continual accumulation of particle-laden AMsΦ, dying and dead cells resulting from PANoptosis, nuclear and cytoplasmic debris, and neutrophilic inflammation. Furthermore, there is buildup of large numbers of T and B cells, along with IgG-secreting plasma cells, suggestive of ectopic lymphoid tissue (ELT). Consistent with prolonged particle-induced pulmonary inflammation and ELT formation, lung fluid and blood from cSiO_2_-instilled mice have elevated proinflammatory cytokines, chemokines, and autoantibodies. As illustrated in Figure 7, these observations support the lung playing an essential role as the nexus for cSiO_2_-induced systemic autoimmune flaring and glomerulonephritis in the NZBWF1 mouse.

A potential promising intervention against cSiO_2_-induced chronic lung inflammation and resultant autoimmunity is increasing dietary intake of the marine polyunsaturated fatty acids (PUFAs) docosahexaenoic acid (C22:6 ω-3; DHA) and eicosapentaenoic acid (C20:5 ω-3; EPA) (Wierenga et al., 2019). Modes of action for ω-3 PUFAs’ ameliorative effects include 1) moderating membrane and lipid raft function, 2) up- and down-regulating gene expression, 3) competing with ω-6 PUFAs and their downstream proinflammatory eicosanoids, and 4) pro-resolving actions of their downstream metabolites [reviewed by Akbar et al. (2017), Calder (2017), Ferreira et al. (2019), and Wierenga et al. (2020)]. Preclinical (Halade et al., 2010; Halade et al., 2013; Pestka et al., 2014) and clinical investigations (Akbar et al., 2017; Li et al., 2019b; Charoenwoodhipong et al., 2020; Duarte-Garcia et al., 2020) indicate that ω-3 PUFAs can counter onset and progression of lupus symptoms, including nephritis. We have found that dietary DHA supplementation reflecting realistic human consumption (i.e., 2 and 5 g/d) can be employed as a prophylactic approach against cSiO_2_-triggered autoimmune flaring in NZBWF1 mice (Bates et al., 2018). DHA consumption specifically inhibited cSiO_2_-triggered pulmonary accumulation of B and T cells, follicular DCs, and IgG^+^ plasma cells. Importantly, DHA dose-dependently inhibited cSiO_2_-triggered lung mRNA signatures indicative of inflammation-, chemokine-, and interferon (IFN)-related gene pathways (Benninghoff et al., 2019). Additionally, DHA supplementation suppresses both cSiO_2_-induced autoantibody responses against a large number of SLE-associated autoantigens (Rajasinghe et al., 2020) and cSiO_2_-triggered glomerulonephritis (Bates et al., 2018). Lastly, we have recently demonstrated that DHA supplementation has value as a therapeutic intervention in this model (Pestka et al., 2021). The demonstration that DHA acts at many stages of cSiO_2_-induced autoimmunity (Figure 7) raises the possibility that ω-3 PUFA supplementation could be used as an intervention against other diseases associated with particle-triggered inflammation and autoimmunity.

## Conclusions and Future Directions

Particle toxicology is a longstanding research field with origins in the 15th century. While this field primarily focused on toxic impacts of inhaled particles in the lung and their connections to occupational disease, it now encompasses a much broader arena that includes seeking to understand how exogenous and endogenous particles influence development of inflammatory and autoimmune diseases in diverse organs. Interestingly, the mechanisms by which particles trigger autoimmunity align with Polly Matzinger’s danger model, which argues that ongoing production and insufficient clearance of danger signals contributes to autoreactivity. Some outstanding knowledge gaps in the field of particle toxicology include understanding how genetics influence the immunotoxic potential of particles, how particles impact other immune cell populations (e.g., innate lymphoid cells, natural killer cells), and how particle toxicology studies can be performed *in silico* to assess risks associated with an individual’s environment and lifestyle. Answering these questions will lead to new understanding of the mechanisms by which particles elicit toxicity in the context of the genome, and will provide valuable insight into new interventions that can be used to prevent or treat particle-associated inflammatory and autoimmune diseases.

## Abbreviations

alum, aluminum-containing salts; AMΦ, alveolar macrophage; BMDMΦ, bone marrow-derived macrophage; CaOx, calcium oxalate; CaP, calcium phosphate; CC, cholesterol crystal; CNT, carbon nanotube; DAMP, damage-associated molecular pattern; DC, dendritic cell; KC, Kupffer cell; MSU, monosodium urate; NET, neutrophil extracellular trap; PAMP, pattern-associated molecular pattern; ROS, reactive oxygen species; SiO_2_, silicon dioxide; TiO_2_, titanium dioxide; TLR, toll-like receptor.
